# Neuronal SPI1 suppression enhances axonal regeneration after spinal cord injury through Rassf10 downregulation

**DOI:** 10.1093/burnst/tkag054

**Published:** 2026-08-13

**Authors:** Dexia Kong, Roujia Kong, Yingying Yan, Haokun Zheng, Xiaowei Qian, Xu Chen, Yan Liu, Mei Liu, Ronghua Wu

**Affiliations:** Jiangsu Key Laboratory of Tissue Engineering and Neuroregeneration, Key Laboratory of Neuroregeneration of Ministry of Education, Co-innovation Center of Neuroregeneration, Nantong University, 19 Qixiu Road, Chongchuan District, Nantong, Jiangsu 226001, China; Jiangsu Key Laboratory of Tissue Engineering and Neuroregeneration, Key Laboratory of Neuroregeneration of Ministry of Education, Co-innovation Center of Neuroregeneration, Nantong University, 19 Qixiu Road, Chongchuan District, Nantong, Jiangsu 226001, China; Research Center of Clinical Medicine, Affiliated Hospital of Nantong University, 20 Xisi Road, Chongchuan District, Nantong, Jiangsu 226001, China; Department of Pediatrics, Medical School of Nantong University, 19 Qixiu Road, Chongchuan District, Nantong, Jiangsu 226001, China; Jiangsu Key Laboratory of Tissue Engineering and Neuroregeneration, Key Laboratory of Neuroregeneration of Ministry of Education, Co-innovation Center of Neuroregeneration, Nantong University, 19 Qixiu Road, Chongchuan District, Nantong, Jiangsu 226001, China; Jiangsu Key Laboratory of Tissue Engineering and Neuroregeneration, Key Laboratory of Neuroregeneration of Ministry of Education, Co-innovation Center of Neuroregeneration, Nantong University, 19 Qixiu Road, Chongchuan District, Nantong, Jiangsu 226001, China; Jiangsu Key Laboratory of Tissue Engineering and Neuroregeneration, Key Laboratory of Neuroregeneration of Ministry of Education, Co-innovation Center of Neuroregeneration, Nantong University, 19 Qixiu Road, Chongchuan District, Nantong, Jiangsu 226001, China; Jiangsu Key Laboratory of Tissue Engineering and Neuroregeneration, Key Laboratory of Neuroregeneration of Ministry of Education, Co-innovation Center of Neuroregeneration, Nantong University, 19 Qixiu Road, Chongchuan District, Nantong, Jiangsu 226001, China; Jiangsu Key Laboratory of Tissue Engineering and Neuroregeneration, Key Laboratory of Neuroregeneration of Ministry of Education, Co-innovation Center of Neuroregeneration, Nantong University, 19 Qixiu Road, Chongchuan District, Nantong, Jiangsu 226001, China

**Keywords:** SPI1, Rassf10, Axonal outgrowth, p38 MAPK, Spinal cord injury

## Abstract

**Background:**

Axonal regeneration following spinal cord injury (SCI) is hindered by the intrinsic inhibitory properties of spinal neurons. While the role of the transcription factor SPI1 (PU.1) has been well established in myeloid cells, its function in neurons, particularly in regulating axonogenesis, remains poorly defined. The data from recent single-cell sequencing studies have indicated that SPI1 is clearly expressed in spinal neurons. Thus, we aimed to explore the effect of SPI1 on axonal growth and its underlying molecular mechanisms.

**Methods:**

Primary cultured rat spinal neurons, CRISPR/Cas9-mediated SPI1 mutation in zebrafish, and neuron-specific AAV9–shRNA delivery after rat spinal cord hemisection were utilized to manipulate SPI1 expression. RNA-seq, luciferase reporter assays, ChIP–qPCR, and Cut&Tag assays were employed to identify and validate Rassf10 as a candidate target gene of SPI1. Axonal outgrowth and functional recovery postinjury were assessed both *in vitro* and *in vivo*.

**Results:**

Neuronal SPI1 expression declined progressively during spinal cord development but was markedly upregulated after SCI. SPI1 knockdown promoted axonal outgrowth, whereas SPI1 overexpression significantly reduced the axonal length. The transcriptomic analysis revealed that Rassf10 is a target gene of SPI1, which was subsequently validated by the results of luciferase reporter, ChIP–qPCR, and Cut&Tag assays. *In vivo*, spi1 mutation in zebrafish and neuron-specific SPI1 knockdown in rats facilitated axonal regeneration and functional recovery after SCI, as evidenced by significant increases in GAP43, NF200, and 5-HT expression and improved motor function compared with the control animals. The depletion of SPI1 or Rassf10 increased p-p38 mitogen-activated protein kinase levels and promoted axonal growth, whereas p38 inhibition abrogated these effects.

**Conclusions:**

SPI1 impairs axonal growth and regrowth by transcriptionally activating Rassf10 expression, with this inhibitory effect mediated by the suppression of p38 MAPK signaling. These findings suggest that targeting the SPI1–Rassf10 pathway represents a promising therapeutic strategy for improving neural repair after SCI.

## Graphical Abstract

**Graphical Abstract:**
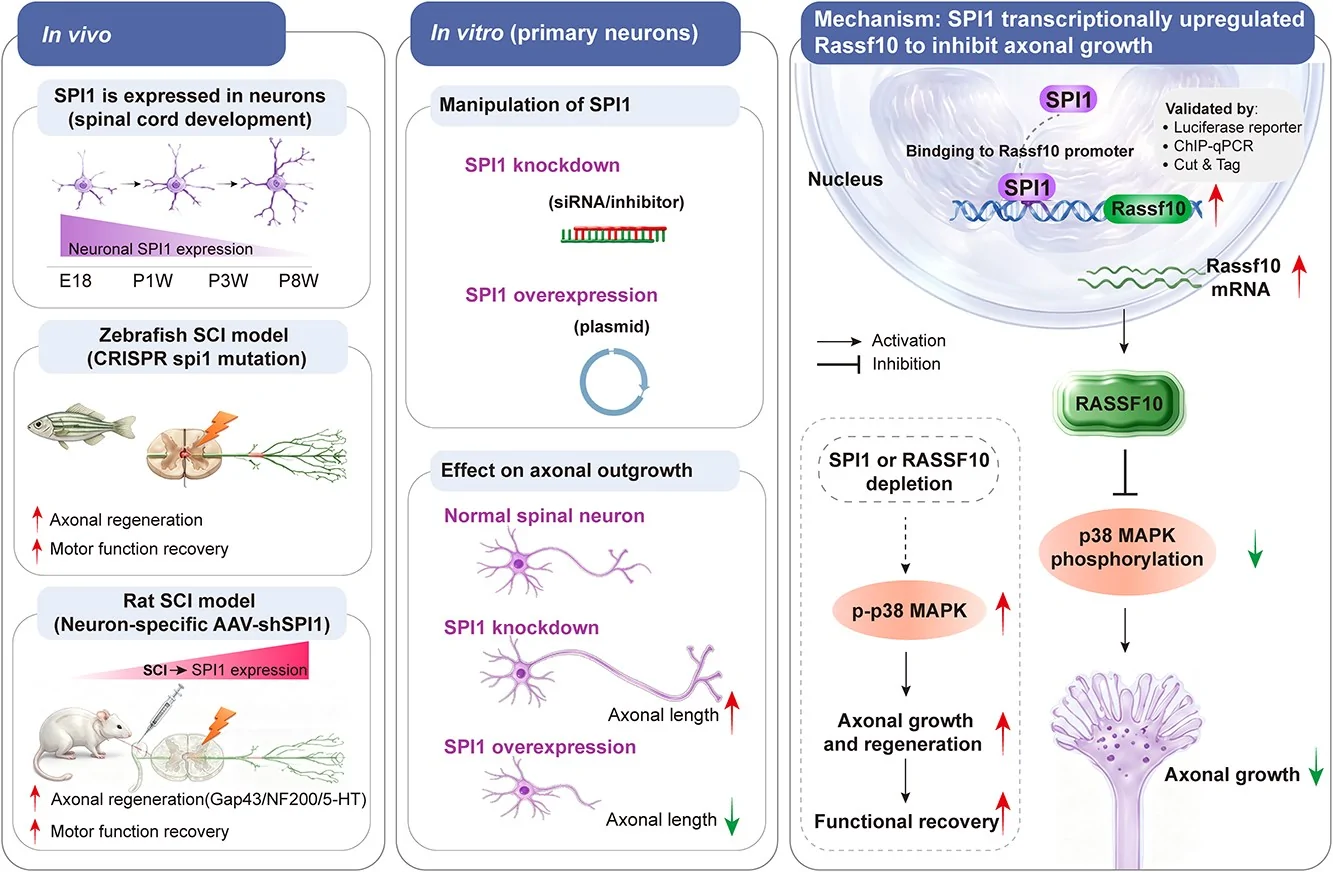

## Highlights

- This study identifies a novel nonimmune function of SPI1 in neurons; its dynamic expression (characterized by gradual downregulation in neurons during development and a shift in its subcellular localization) serves as a key negative regulator of axonal growth.
- The regulatory mechanism of the SPI1–Rassf10 axis was elucidated: SPI1 directly binds to the Rassf10 promoter and modulates axonal growth by regulating the p38 mitogen-activated protein kinase pathway.
- The results of cross-species validation experiments confirm that SPI1 is a therapeutic target for spinal cord injury and that SPI1 knockdown in zebrafish/rat models enhances both axon regeneration and functional recovery.

## Background

Axonal outgrowth is essential for central nervous system (CNS) development and plays a pivotal role in neuronal function and regeneration after injury [1, 2]. Despite significant progress, the complex mechanisms that regulate axon growth, guidance, and regeneration remain inadequately understood. Identifying key regulatory factors is critical for advancing therapeutic strategies designed to promote axonal regeneration and functional recovery. Transcription factors orchestrate gene expression programs critical for cellular differentiation and development [3, 4]. Although transcription factors such as c-Jun are well-established regulators of axon growth [5], identifying novel molecules that regulate axon growth may pave the way for the development of new therapeutic approaches to promote neurological repair.

SPI1 (also known as PU.1) is a well-established master regulator of myeloid cell differentiation and development [6, 7]. Emerging studies have begun to elucidate its broader roles in the nervous system, and the results suggest that it has functions beyond immune cell regulation [8–10]. However, the research that has been published to date has focused predominantly on microglia and macrophages. Preliminary data from our laboratory revealed dynamic SPI1 expression in spinal cord neurons during development: pronounced nuclear localization at embryonic stages, followed by a progressive decline and a shift to a cytoplasmic localization during the postnatal period. Notably, neuronal SPI1 expression was upregulated following spinal cord injury (SCI), concomitant with increased nuclear translocation of the SPI1 protein. Additionally, single-cell data from spinal cord tissues revealed significant SPI1 expression in neurons [11]. The manipulation of SPI1 expression in spinal cord neurons revealed that its knockdown significantly increased the axon length, whereas its overexpression inhibited axonal growth. These findings suggest that SPI1 regulates neuronal processes and that the molecular mechanisms underlying its effects on axon growth warrant further investigation.

Rassf10, a tumor suppressor gene that is epigenetically silenced in cancers and a candidate downstream target of SPI1, has been implicated in various cellular processes, including cell proliferation and apoptosis [12, 13]. However, its role in neuronal processes remains unexplored. Furthermore, the p38 mitogen-activated protein kinase (MAPK) pathway, a key mediator of stress responses and axonal growth [14–16], represents a potential downstream effector of SPI1–Rassf10 signaling.

In this study, a novel SPI1-dependent axis that regulates axonal outgrowth via Rassf10 was identified. Using CRISPR/Cas9-mediated gene editing in zebrafish, transcriptomic profiling, functional assays in primary neurons, and neuron-specific knockdown in a rat spinal cord hemisection model, this study demonstrated that SPI1 transcriptionally upregulates Rassf10, thereby attenuating p38 MAPK phosphorylation and suppressing axonal outgrowth. *In vivo*, SPI1 knockdown in rats promoted axonal regeneration and functional recovery after SCI, as evidenced by increased GAP43, NF200, and 5-HT expression and improved motor function. These findings redefine SPI1 as a dual regulator of both immune responses and neuronal plasticity, providing a mechanistic basis for targeting the SPI1–Rassf10 axis in neurological disorders.

## Materials and Methods

### Analysis of single-cell data

Single-cell ribonucleic acid sequencing (RNA-seq) profiles of injured mouse spinal cord tissues were retrieved from the Gene Expression Omnibus (GEO) database (accession GSE172167) [11]. Data integration was performed using R v4.1.0, and downstream visualization and statistical analyses were conducted using Seurat v4.4.1, ggplot2 v3.5.2, ggthemes v5.1.0, and dplyr v1.1.4. Cells were clustered and assigned to major CNS cell types based on canonical marker gene expression. SPI1 transcript levels were extracted for each cluster and visualized in violin plots.

### Ribonucleic acid extraction and real-time quantitative polymerase chain reaction

Total RNA was isolated from collected samples via the RNA-Quick Purification Kit (Cat# ES-RN001; Yibin Bio) following standard nucleic acid extraction procedures [17]. Reverse transcription for complementary deoxyribonucleic acid (cDNA) synthesis was carried out with the HiScript III 1st-Strand cDNA Synthesis Kit (+ gDNA wiper) (Cat# R312-02, Vazyme). Gene-specific primer pairs were applied in polymerase chain reaction (PCR) reactions, where 18S rRNA served as an internal reference to verify intact mRNA quality. Quantitative real-time PCR (qPCR) amplification was implemented on the qTower^3^G platform (Analytik Jena, Germany) using AceQ qPCR SYBR Green Master Mix (High ROX, Cat# q142-02, Vazyme). All target primer sequences, manufactured by GENCEFE Biotech (Wuxi, China), have been summarized in Table 1.

**Table 1:** The sequences of the primers, siRNAs and sgRNAs used in the study

Name	Sense (5’ - 3’)	Antisense (5’ - 3’)
18S rRNA	ggacacggacaggattgaca	caatctcgggtggctgaac
Rassf10	tgcgtacttttgagcagacg	tggagctcagcccagtatct
Spi1-1	atggagacagccatagcgatca	ggtgtcaaggacgtgcatct
Spi1-2	ctccatcggatgagctggtt	cagctccgtgaagtggttct
Spi1-Rassf10-ChIP binding	ggcacaagaccacacacatc	ctcaccaggtatcagctctgc
ChIP non-binding	tgggacctcagccaatgttc	ctcaagaccttggatgcctca
Control (Ctrl) siRNA	uucuccgaacgugucacgutt	acgugacacguucggagaatt
(r) Rassf10-siRNA	gcgaaggcauggagucaactt	guugacuccaugccuucgctt
(r) Spi1-siRNA	gaacuauaccaacgucaaatt	uuugacguugguauaguuctt
Spi1a sgRNA	(mA)*(mU)*(mA)*CGGGUAGAAGUCGUAUAGUUUUAGAGCUAGAAAUAGCAAGUUAAAAUAAGGCUAGUCCGUUAUCAACUUGAAAAAGUGGCACCGAGUCGGUGCU*(mU)*(mU)*(mU)	
Spi1b sgRNA	(mG)*(mC)*(mU)*GCAUCCGUACAGAAUGGGUUUUAGAGCUAGAAAUAGCAAGUUAAAAUAAGGCUAGUCCUUAUCAACUUGAAAAAGUGGCACCGAGUCGGUGCU*(mU)*(mU)*(mU)	

### Western blot analysis

Cell lysates were harvested to extract total cellular protein, whose concentrations were quantified via a bicinchoninic acid assay kit following standard immunoblot protocols [18]. Equal-volume protein aliquots (10–20 μg) underwent separation through sodium dodecyl sulfate–polyacrylamide gel electrophoresis, then electrotransferred onto polyvinylidene difluoride membranes under a steady current of 200 mA. Membranes were blocked with 5% non-fat skim milk diluted in TBST for 2 h at ambient temperature, then probed overnight with primary antibodies as listed below: SPI1 (Cat# 66618-2-Ig, Proteintech, 1:1000), p-p44/42 MAPK (Cat# 4370, CST, 1:1000), total p44/42 MAPK (Cat# 4695 T, CST, 1:1000), total p38 MAPK (Cat# 8690, CST, 1:1000), p-p38 MAPK (Cat# 4511 T, CST, 1:1000), β-actin (Cat# 60008-1-Ig, Proteintech, 1:1000), GAPDH (Cat# 60004-1-Ig, Proteintech, 1:1000). After three TBST rinses, blots were incubated with horseradish peroxidase-linked anti-mouse or anti-rabbit secondary antibodies for 2 h at room temperature, followed by another three rounds of TBST washing. Protein bands were visualized with chemiluminescent substrate (Cat# P10300, NCM Biotech) and captured by a Tanon 5200 imaging system (Tanon, China). Band intensity was analyzed using Multi Gauge software (Fujifilm, Japan), with target protein signals normalized to endogenous GAPDH or β-actin loading controls.

### Immunohistochemistry

For immunohistochemical staining [17, 19], rats underwent transcardial perfusion with 4% paraformaldehyde at predetermined time points. Segments of T10 spinal cord were post-fixed in the same fixative overnight at 4°C, then cryoprotected in 30% sucrose for 24 h and preserved at −20°C. A cryostat was used to prepare 16 μm-thick transverse tissue slices. Sections were incubated overnight at 4°C with the following primary antibodies: anti-PU.1 polyclonal antibody (Cat# PA5-80066, Invitrogen, 1:400), NeuN (Cat# ab104224, Abcam, 1:400), Iba1 (Cat# ab283346, Abcam, 1:400), GAP43 (Cat# 16971-1-AP, Proteintech, 1:400), GFAP (Cat# ab4674, Abcam, 1:1000), NF200 (Cat# N4142, Sigma, 1:400), and 5-HT (Cat# 20079, Immunostar, 1:1000). After three phosphate-buffered saline (PBS) rinses, slices were reacted with secondary antibodies for 2 h at ambient temperature. Cell nuclei were counter-labeled with 4′,6-diamidino-2-phenylindole (DAPI) for 15 min, followed by another three PBS washes. Fluorescent signals were acquired via an LSM 900 Airyscan 2 super-resolution confocal microscope.

### Primary neuron culture

Primary spinal neurons were isolated from E14 embryos of pregnant Sprague-Dawley (SD) rats as previously reported [18]. Briefly, pregnant rats were anesthetized and sacrificed to collect embryonic spinal cords in Hank's balanced salt solution. Tissues were cut into small fragments with micro scissors and digested in 0.125% trypsin for 30 min; digestion was terminated with Dulbecco's Modified Eagle Medium/F12 medium containing 10% fetal bovine serum, 1% glutamine and triple antibiotics. Dissociated cells were triturated into single-cell suspension, filtered through a 40 μm Nitex strainer and pelleted by centrifugation at 1200 rpm for 5 min. Cell precipitates were resuspended in neuron culture medium supplemented with 2% NeuroCult™ SM1 (Cat# 5711, STEMCELL), 1× L-glutamine (Cat# C0212, Beyotime) and triple antibiotics, then cultured at 37°C under humidified 95% air/5% CO₂.

### Cell transfection

Electroporation mediated by the NEPA21 device was used to transfect E14 spinal neurons with small interfering RNA (siRNAs) at a terminal concentration of 200 nM [18, 20]. All siRNA fragments and overexpression plasmids were chemically synthesized by GENCEFE Biotech (Wuxi, China), with full sequences listed in Table 1. In brief, 3 × 10^6^ neuronal cells were resuspended in 90 μl Minimum Essential Medium (MEM), blended with 10 μl siRNA or plasmid solution, and electroporated under parameters of 275 V and 0.7 ms pulse duration. Post-electroporation cells were re-suspended in complete neuronal medium and seeded into 6-well culture plates. The medium was refreshed 6 h later once cells fully attached to the plate bottom.

### Immunocytochemistry

For immunocytochemistry [18], *in vitro*-cultured E14 primary neurons were fixed in 4% paraformaldehyde and blocked with blocking solution at 37°C for 1 h. Cells were incubated overnight at 4°C with purified mouse anti-TUBB3 antibody (Cat# 801202, BioLegend, 1:800). After three PBS washes, samples were treated with Alexa Fluor 488-labeled goat anti-mouse secondary IgG (1:400) for 2 h, and cell nuclei were counterstained with DAPI over 15 min. Fluorescent pictures were captured via a Leica inverted fluorescence microscope. Consistent with our prior work [21], neurites longer than 20 μm were classified as axons in subsequent quantitative analysis.

### Single-guide RNA synthesis and zebrafish embryo microinjection

Single-guide RNAs (sgRNAs; sequences detailed in Table 1) were synthesized by Tsingke Biotechnology Co., Ltd, and the Cas9 protein was purchased from GenScript Biotech Corporation (Cat# Z03388). The embryos were collected and rinsed with E3 buffer and then arranged in a mold slot. The injection solution was drawn into a glass capillary tip equipped with an Eppendorf micropipette tip. The capillary was mounted onto a ChengMao IM400 microinjector, and the microscope focus was adjusted to clearly visualize the injection needle. The injection pressure and volume were carefully controlled using the microscope's injector panel, with a standard volume of 1 nanoliter (nL) per embryo.

### Zebrafish behavioral assays

Individual zebrafish were placed in separate wells of a 24-well plate and monitored using EthoVision XT 13 (Noldus Information Technology). Following a 10-min acclimation period to ensure the normalization of behavior, the fish were tracked continuously for 30 min. The system's software was subsequently employed for the automated analysis and processing of the behavioral data [22].

Before the experiment, the zebrafish from both groups were transferred to fresh E3 buffer in Petri dishes and allowed to acclimate for 30 min to minimize stress. Tail flick movements were recorded at 4 days post-injury using a high-speed camera, with each recording lasting 3 min. The temperature was maintained at 28.5°C throughout the experiment to prevent temperature-induced artifacts. The recorded behaviors included spontaneous tail bending, forward swimming, and turning. For the quantitative analysis, a 10-second segment was extracted from each video to measure the maximum tail bending amplitude at the injury plane, which was defined as the peak deflection angle of the tail tip [23].

### Establishment of the zebrafish tissue injury model

Zebrafish larvae at 72 hours post-fertilization (hpf) were anesthetized using 40 mg/L tricaine for 15 min to ensure complete anesthesia. Following anesthesia, the larvae were placed on a 2% agarose gel and subjected to SCI at the level above the yolk extension using a spinal cord hemisection knife. The injured zebrafish were then placed in E3 buffer supplemented with ampicillin (1:1000) and 0.003% phenylthiourea. After a 24-h recovery period, the larvae were re-anesthetized and fixed in 0.5% low-melting-point agarose. Fluorescence images were captured using a high-speed super-resolution confocal microscope (LSM 900 with Airyscan 2). After imaging, each zebrafish was carefully removed from the 0.5% agarose gel. Genomic DNA was extracted using YSY Buffer for lysis, followed by PCR amplification. The PCR products were subsequently sent to Genewiz (Suzhou) Co., Ltd, for sequencing analysis.

### Luciferase reporter assay and chromatin immunoprecipitation

For dual-luciferase reporter detection, HEK293T cells were co-transfected via Lipofectamine 8000 transfection reagent (Cat# C0533, Beyotime), with all recombinant plasmids manufactured by GENCEFE Biotech (Wuxi, China). At 48 h after transfection, luminescence signals were quantified with the Dual Luciferase Reporter Assay Kit (Cat# MA0518-1, MeilunBio®); Renilla luciferase signals acted as an internal standard to calibrate firefly luciferase readings [17, 24].

Chromatin immunoprecipitation (ChIP) was implemented following the official instructions of the SimpleChIP® Enzymatic Chromatin IP Kit (Magnetic Beads, Cat# 9005, CST) [25, 26]. Briefly, primary E14 spinal neurons isolated from SD rat embryos were fixed in formaldehyde to create covalent crosslinks between DNA and chromatin proteins, then fragmented into 150–900 bp fragments via micrococcal nuclease digestion. Chromatin complexes bound by anti-histone or anti-SPI1 antibodies were captured with Protein G magnetic beads. After crosslink reversal, immunoprecipitated DNA was purified for downstream detection. Purified DNA templates were subjected to qPCR amplification on a qTower^3^G platform (Analytik Jena, Germany) using AceQ qPCR SYBR Green Master Mix (High ROX, Cat# q142-02, Vazyme). Finally, 2 μl aliquots of PCR products were separated on 2% agarose gel for electrophoretic validation.

### RNA sequencing and bioinformatics analysis

Total RNA was isolated from neurons transfected with control siRNA and SPI1-targeted siRNA, then purified for library construction with the TruSeq Stranded mRNA LT Kit (Illumina, San Diego, CA, USA) following the manufacturer's standard protocols [17]. All libraries underwent Illumina sequencing to acquire raw imaging signals, which were converted into FASTQ raw sequencing reads by platform-specific software. Raw datasets containing residual adapters and low-quality sequences were trimmed via fastp v0.22.0 to yield high-quality clean reads for subsequent bioinformatic processing.

Gene-level read counting was accomplished with HTSeq v0.9.1, and transcript abundance was normalized into FPKM values for cross-sample comparison. Differential expression analysis was carried out with DESeq v1.38.3, where genes satisfying |log₂FC| > 1 and *P* < .05 were defined as differentially expressed genes (DEGs). The topGO v2.50.0 package was adopted to conduct Gene Ontology (GO) enrichment for total, upregulated and downregulated DEGs, with statistical *P*-values computed based on hypergeometric distribution (threshold: *P* < .05). Kyoto Encyclopedia of Genes and Genomes (KEGG) pathway enrichment was implemented through clusterProfiler v4.6.0, and pathways with *P* < .05 were regarded as significantly enriched (Supplementary Table S1).

### Cut&Tag

Cut&Tag was performed using the Hyperactive Universal Cut&Tag Detection Kit for Illumina (Cat# TD904, Vazyme) according to the manufacturer's instructions [27]. Briefly, 1 × 10^5^ spinal neuronal cells were immobilized on ConA-coated magnetic beads and subsequently incubated with an anti-SPI1 primary antibody, rabbit anti-mouse IgG, and pA/G-Tn5 transposase (0.04 μM). After being washed, the samples were incubated for 1 h at 37°C in Dig-300 buffer containing 5 × TTBL. The reaction was terminated with 10% SDS. DNA fragments were extracted using DNA Extraction Beads Pro, and Stop Buffer was added to elute the DNA. The resulting fragmented DNA was subjected to real-time quantitative PCR using AceQ qPCR SYBR Green Master Mix (High ROX, #q142-02, Vazyme) on a qTower^3^G system (Analytik Jena, Germany).

Libraries were amplified for 12 cycles using the TruePrep Index Kit and sequenced on an Illumina NovaSeq 6000 system (PE150). The complete sequencing workflow was performed by Personal Biotechnology Co., Ltd (Shanghai, China). The results of the post-sequencing analysis are presented in Supplementary Table S2.

Target gene selection was performed by intersecting three complementary sequencing datasets (Supplementary Table S2): (i) developmentally upregulated genes from the time course analysis conducted at E18, P1, and P3 (correlating with SPI1 expression), (ii) SPI1-bound genes identified by Cut&Tag (SPI1 *vs* IgG), and (iii) genes that were downregulated following SPI1 silencing (siSPI1 *vs* siCtrl).

### Adeno-associated virus injection

Adeno-associated virus (AAV) vectors carrying the neuron-specific synapsin (Syn) promoter were used to achieve neuron-specific knockdown of SPI1 in the spinal cord. The recombinant AAV9–Syn–EGFP–shSPI1 virus (3.59 × 10^12^ vg/ml; Obio Technology Corp., Ltd, Shanghai) or a scrambled control (AAV9–Syn–EGFP–shCtrl; 3.63 × 10^12^ vg/ml) was stereotaxically injected into the T8 spinal cord of adult SD rats (200–220 g). Under 2% isoflurane anesthesia, a dorsal laminectomy was used to expose the spinal cord, and a glass micropipette (tip diameter ≈ 20 μm) was inserted through the dura mater. Two injection sites were used (Fig. 4a), and 1 μl was injected at each point at each site; 0.34 μl was injected at 1.5 mm under the spinal dura mater, and the needle was then slowly lifted by 1.2 mm to inject 0.33 μl and finally lifted by 0.9 mm to inject the residual 0.33 μl [17]. The total injection volume of 2 μl was infused at a flow rate of 0.5 μl per minute, followed by a 2-min retention of the micropipette at the injection site to reduce liquid backflow.

### Rat SCI

T10 lateral hemisection was performed as described previously [17, 28, 29]. Under 2% isoflurane anesthesia, a dorsal midline incision was made from T9–T10, and the paraspinal muscles were retracted to expose the vertebrae. A complete laminectomy was performed at T10, with the dura mater left intact. The right hemicord was transected with a microfeather scalpel from the dorsal median sulcus to the ventral root exit zone. Hemostasis was achieved with sterile saline-soaked Gelfoam. Muscles and skin were closed in layers, and postoperative care included manual bladder expression and prophylactic ceftriaxone (100 mg kg^−1^ day^−1^) for 3 days. Postoperatively, all rats were single-housed within a climate-regulated rearing chamber (22 ± 1°C, 50%–60% relative humidity) maintained on a 12 h light/dark photoperiod, with unlimited access to standard chow and drinking water. Throughout the experimental period, animal physical conditions, body weight, infection signals and abnormal stress responses were inspected twice per day.

All animal experiments described in this manuscript were reviewed and approved by the Animal Care and Use Committee of Nantong University under ethical approval license P20250228-038*.*

### Behavioral assessment

Locomotor functional restoration in the open field was quantified via the Basso–Beattie–Bresnahan (BBB) rating scale [30, 31]. This scoring system ranges from 0 (total hindlimb paralysis) to 21 (fully intact motor performance). Every behavioral evaluation was conducted under strict double-blind experimental conditions. Observers were excluded from surgical procedures and remained blinded to the group allocation, treatment conditions, and animal identity. Each rat was assigned a random code, and scoring was conducted in a separate room. Two independent, trained observers performed the evaluations independently, and their average scores were used for the statistical analysis. Blinding was maintained throughout data analysis.

The CatWalk gait analysis was performed using the CatWalk XT system (Noldus Information Technology) to quantify motor recovery. Briefly, rats were placed on a glass walkway over an illuminated glass plate, and their footprints were captured by a high-speed camera positioned below. The animals were allowed to walk freely across the walkway for at least three uninterrupted runs each. The system automatically recorded gait parameters, including maximum contact area (cm^2^), mean intensity, base of support (distance between hind paws), and print area (cm^2^). All quantitative analyses were completed by a researcher unaware of the group allocation throughout the experiment.

Hargreaves assay was adopted to assess thermal nociception [32]. Rats were placed in transparent plexiglass compartments atop an IITC Model 390 elevated glass platform and habituated for 30 min. Radiant heat was delivered to the plantar mid-region of each hind paw, with automatic recording of paw withdrawal latency (PWL). The results from three trials per paw, separated by ≥5 min, were averaged. A 20 s cutoff prevented tissue damage.

Terminal electrophysiological assessment was performed at 2 weeks post-injury using a Keypoint 2 portable electromyograph (Dantec, Denmark), as previously described [33]. Under deep anesthesia and thermal maintenance, the spinal cord was re-exposed and the injury site was identified by the nylon suture on the dura. Bipolar tungsten stimulating electrodes (1 mm apart) were placed intraspinally at T8 (rostral) or T12 (caudal) relative to the injury epicenter. The spinal cord was sequentially stimulated with brief square wave pulses (0.2 ms, 600–800 μA) delivered at 3–5 s intervals. Evoked responses were recorded from the gastrocnemius (GAS) muscle using the tendon–belly method, with the active electrode in the mid-belly and the reference electrode in the Achilles tendon. Recordings were repeated three times and averaged.

### Statistical analysis

The data are presented as the means ± standard deviations (SDs). Statistical analyses were performed using GraphPad Prism software (version 10.1.2; GraphPad Software, San Diego, CA, USA). The statistical significance of differences between datasets was tested using an unpaired two-tailed Student's *t* test. One-way analysis of variance (ANOVA) with Tukey's or Bonferroni post hoc correction were used to compare data from multiple groups, and two-way ANOVA with Bonferroni post hoc correction were used when the data were influenced by two factors. Axon lengths were classified into three grades (20–50, 50–100, >100 μm) as described previously [18]. Fisher's exact test was performed on 2 × 3 contingency tables to analyze differences in the axon length distribution between the experimental groups (Fig. 2e and h). Statistical significance was set at *P* <0.05.

## Results

### Developmental dynamics of SPI1 expression in spinal cord neurons and glia

SPI1 (PU.1) is traditionally regarded as a key transcriptional regulator of myelopoiesis, which is essential for the development, differentiation, and immune competence of microglia, the resident myeloid cells of the CNS. A publicly available mouse single-cell RNA-seq dataset [11] (GEO accession: GSE172167) was analyzed to profile the cell type-specific expression of SPI1 in spinal cord tissue. Consistent with its known role, SPI1 transcripts were most abundant in microglia. Notably, however, substantial SPI1 expression was detected in the neuronal clusters (Supplementary Fig. S1a). Neuronal SPI1 expression was upregulated following SCI, accompanied by increased nuclear translocation of the protein (Supplementary Fig. S1b). An analysis of transcriptomic data from the developing cortex [34], which revealed a decline in regenerative potential at embryonic day 18 (E18), postnatal day 1 (P1), and postnatal day 3 (P3), revealed a progressive upregulation of SPI1 expression (Supplementary Fig. S1c). Together, these results reveal an unreported nonimmune function of SPI1 in the CNS. Given its dual expression pattern in immune cells and neurons, SPI1 likely participates in a wider range of neural processes than previously thought, with the capacity to modulate both immune responses and neuronal activity.

Next, the developmental expression of SPI1 was evaluated at key time points, including E18, postnatal week 1 (P1W), postnatal week3 (P3W), and postnatal week 8 (P8W), using quantitative polymerase chain reaction (qPCR) and western blot (WB) analyses (Fig. 1a). The data showed a marked upregulation of SPI1 expression across these stages, with significant increases observed at both the mRNA and protein levels (Fig. 1b and e).

**Figure 1:**
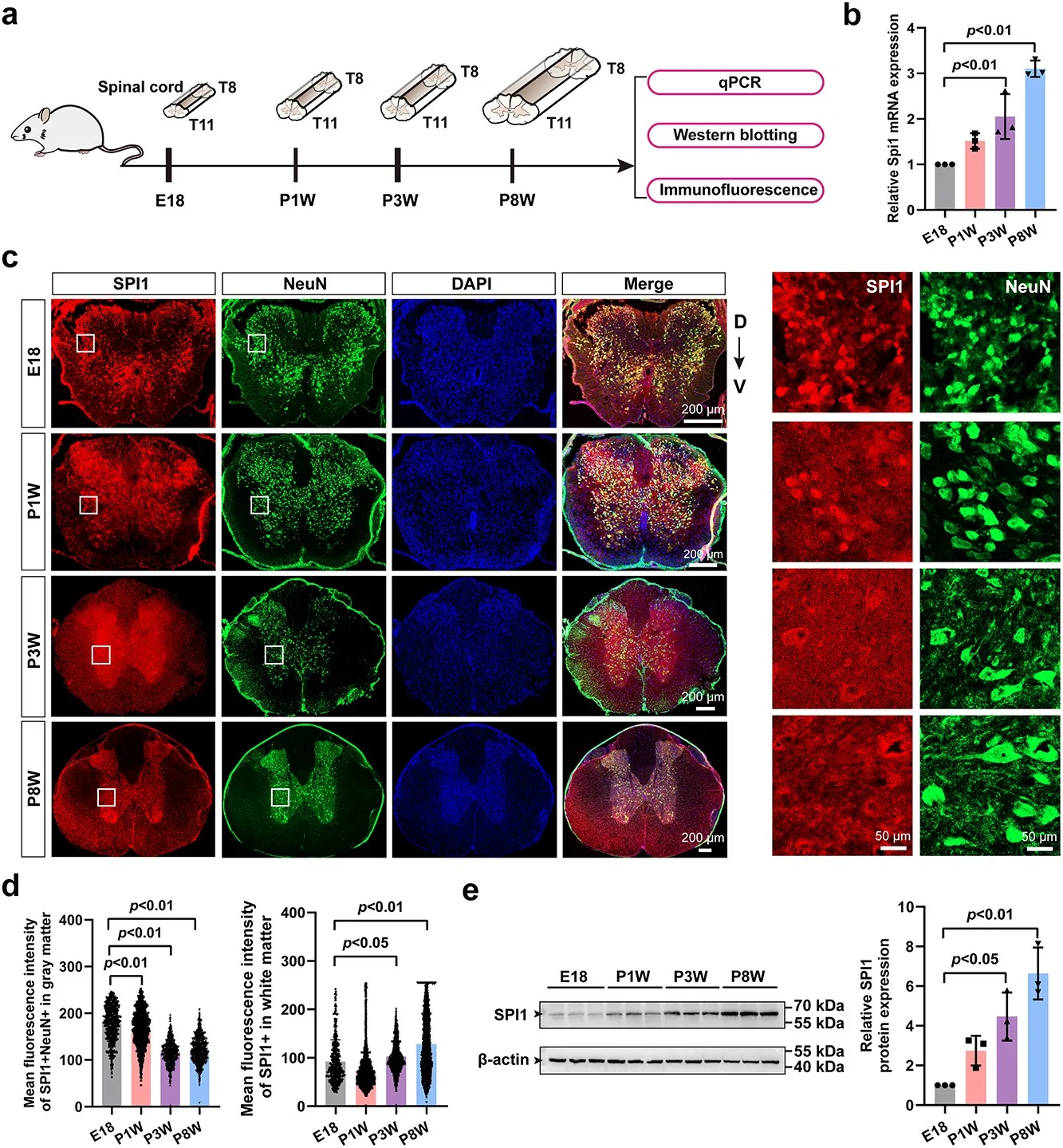
SPI1 dynamics expression during spinal cord development. (**a**) Experimental timeline illustrating the collection of rat spinal cord tissues at E18, postnatal week 1 (P1W), postnatal week 3 (P3W), and postnatal week 8 (P8W) for SPI1 expression analysis. Tissues from segments T8-T11 were subjected to qPCR for mRNA analysis, Western blotting for protein analysis, and IF to visualize SPI1 protein localization. (**b**) qPCR analysis of SPI1 expression at E18, P1W, P3W, and P8W. Data are presented as mean ± SD, *n* = 3 biological replicates. One-way ANOVA followed by Tukey's post hoc test was used for analysis (*P* < 0.05). The *P*-values are indicated in the panel. (**c**) Immunohistochemical (IHC) staining of SPI1 and NeuN in spinal cord tissue at E18, P1W, P3W, and P8W. Left panel shows representative IHC results (scale bar: 200 μm); right panel presents a magnified view of the white box in left panel (scale bar: 50 μm), D, dorsal; V, ventral. (**d**) Statistical analysis of (c). Data are presented as mean ± SD. One-way ANOVA followed by Bonferroni's post hoc test was used for analysis (*P* < 0.05). The *P*-values are shown in the panel. (**e**) SPI1 protein expression at E18, P1W, P3W, and P8W. Left panel displays representative WB results, and right panel provides statistical analysis. Data are presented as mean ± SD, *n* = 3 biological replicates. One-way ANOVA followed by Tukey's post hoc test was used for analysis (*P* < 0.05). The *P*-values are shown in the panel

**Figure 2:**
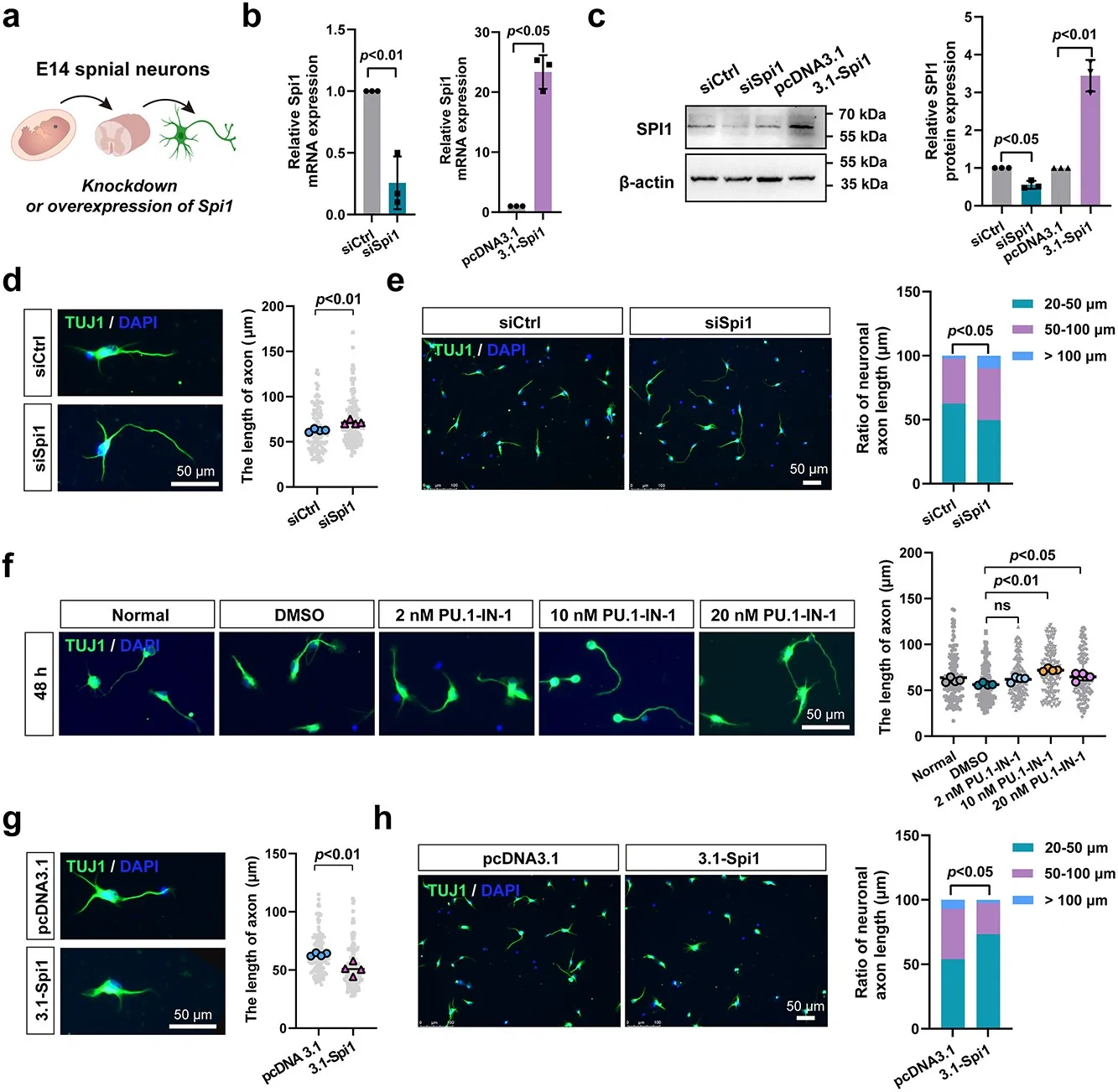
Intervention of SPI1 expression significantly affected axon outgrowth *in vitro*. (**a**) Schematic representation of the experimental setup to investigate the effects of SPI1 on E14 spinal neurons. (**b**) Left panel: the mRNA expression of SPI1 after siRNA treatment in neurons. Right panel: the mRNA expression of SPI1 after overexpression treatment in neurons. Data are presented as mean ± SD, *n* = 3 biological replicates. Unpaired Student's *t-*test was used to compare two groups (*vs* siCtrl/pcDNA3.1). The *P*-values are shown on the panel. (**c**) The protein expression of SPI1 after siRNA or overexpression treatment in neurons. Left panel: representative WB results; right panel: statistical analysis. Data are presented as mean ± SD, *n* = 3 biological replicates. Unpaired Student's *t-*test was used to compare two groups (*vs* siCtrl/pcDNA3.1). The *P*-values are shown on the panel. (**d**) Impact of SPI1 siRNA on axonal growth. Left panel: the representative IF images stained with Tuj1 antibody with a scale bar of 50 μm. Right panel: statistical analysis. Data are presented as mean ± SD, *n* = 4 biological replicates, the gray dots represent 120 neurons. Unpaired Student's *t-*test was used to compare two groups (*vs* siCtrl). The *P*-values are shown on the panel. (**e**) Impact of SPI1 siRNA on the ratio of neuronal axon length. Left panel presents the representative IF images of Tuj1 treated with SPI1 siRNA for 48 h in neurons with a scale bar of 50 μm. Right panel: statistical analysis. (**f**) Impact of PU.1-IN-1 (SPI1 specific inhibitor) on axonal growth. Left panel presents the representative IF images of Tuj1 treated with PU.1-IN-1 (2, 10, 20 nM) for 48 h in neurons with a scale bar of 50 μm. Right panel: statistical analysis. Data are presented as mean ± SD, *n* = 4 biological replicates, the gray dots represent 120 neurons. Data were analyzed using one-way ANOVA followed by Tukey's post hoc test (*P* < 0.05). The *P*-values are shown on the panel. (**g**) Impact of SPI1 overexpression on axonal growth. Left panel: the representative IF images stained with Tuj1 antibody with a scale bar of 50 μm. Right panel: statistical analysis. Data are presented as mean ± SD, *n* = 4 biological replicates, the gray dots represent 120 neurons. Unpaired Student's *t-*test was used to compare two groups (*vs* siCtrl). The *P*-values are shown on the panel. (**h**) Impact of SPI1 overexpression on the ratio of neuronal axon length. Left panel presents the representative IF images of Tuj1 treated with SPI1 siRNA for 48 h in neurons with a scale bar of 50 μm. Right panel: statistical analysis

Immunofluorescence (IF) staining of tissues was performed to further investigate the spatial and temporal dynamics of SPI1 expression. At E18, SPI1 was primarily localized to neurons in the gray matter. During subsequent development, SPI1 expression in these neurons gradually decreased, accompanied by its subcellular redistribution from the nucleus to the cytoplasm. Concurrently, SPI1 expression in glial cells increased, suggesting a role in glial maturation and function (Fig. 1c and d and Supplementary Fig. S1e). These findings highlight the complex and dynamic expression pattern of SPI1 in the developing CNS, revealing its multifaceted effects on both neuronal and glial cell biology.

### The modulation of SPI1 expression markedly influenced axonal outgrowth *in vitro*

E14 spinal cord neurons were cultured *in vitro*, and SPI1 expression was manipulated using siRNA knockdown or overexpression plasmids to further explore its role in neuronal function. qPCR and WB analyses confirmed the efficient knockdown and overexpression of SPI1 at both the mRNA and protein levels (Fig. 2a–c). The effect of SPI1 knockdown on axonal length was subsequently assessed. SPI1 knockdown in these neurons resulted in a significant increase in axonal elongation, with the mean axonal length in the siSPI1 group increasing by 14.4% compared with that in the siCtrl group (71.27 ± 2.476 μm *vs* 62.29 ± 1.618 μm, respectively; Fig. 2d). Neurons were classified into three categories based on the axonal length [18]: 20–50, 50–100, and > 100 μm. As shown in Figure 2e, compared with those in the control group (siCtrl), the ratios of neurons with axon lengths >100 and 50–100 μm was noticeably higher in the siSPI1 group. Furthermore, neurons cultured under standard conditions were treated with various concentrations of the SPI1-specific inhibitor PU.1-IN-1. After 48 h, axonal lengths were measured, and treatment with the inhibitor at 10 and 20 nM concentrations significantly increased axonal growth compared with the control treatment (Fig. 2f). In contrast, SPI1 overexpression markedly reduced the axonal length, with the 3.1-SPI1 group showing a 19.6% decrease compared with the pcDNA3.1 group (50.91 ± 5.559 μm *vs* 63.31 ± 1.475 μm). Moreover, significantly fewer neurons in the overexpression group exhibited axons of 50–100 μm or > 100 μm compared with control cells (Fig. 2g and h).

Collectively, these results establish that SPI1 is a negative regulator of axonal growth, with its expression inversely correlated with the neuronal regenerative capacity.

### Mutation of spi1 in ziebrafish robustly enhanced axonal outgrowth and functional restoration following SCI

To further explore the function of Spi1 in axonal outgrowth, we adopted CRISPR-Cas9-mediated genome-editing to disrupt two spi1 paralogs (spi1a and spi1b) in the transgenic Tg(hb9:eGFP) zebrafish line, in which neuronal axons can be visualized via eGFP fluorescence. Isoform-targeting sgRNAs against spi1a and spi1b were co-injected together with Cas9 ribonucleoprotein into one-cell-stage embryos to generate efficient somatic gene disruption. Target-site mutations were validated via genomic sequencing (Fig. 3a).

**Figure 3:**
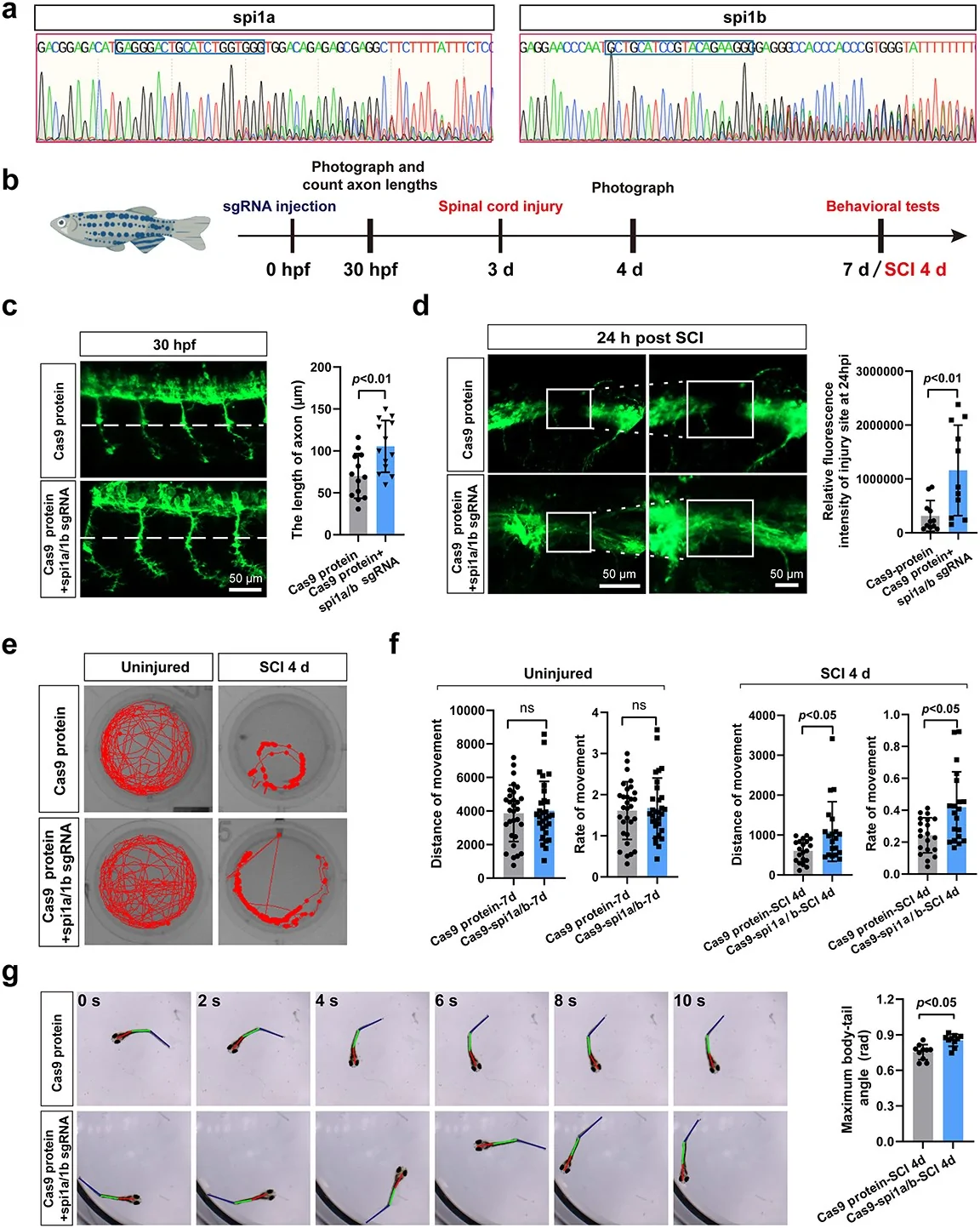
Mutation of spi1 expression in zebrafish significantly promoted axon outgrowth and recovery of function post SCI (**a**) validation of spi1a and spi1b sgRNA efficacy. Left panel: sequencing peak map of the spi1a PCR product; right panel: sequencing peak map of the spi1b PCR product. (**b**) Experimental timeline depicting the manipulation procedures. (**c**) Effects of spi1 mutation on zebrafish axon growth at 30 hours post-fertilization (hpf) using CRISPR/Cas9 technology. Left panel: representative images (scale bar: 50 μm); right panel: statistical analysis. Data are presented as mean ± SD, *n* = 13 zebrafish per group. Unpaired Student's *t-*test was used to compare two groups (*vs* Cas9 protein). The *P*-values are indicated on the panel. (**d**) Effects of spi1 mutation on zebrafish axon growth 24 h post-SCI. Left panel: representative images (scale bar: 50 μm); right panel: statistical analysis. Data are presented as mean ± SD, *n* = 11 zebrafish per group. Unpaired Student's *t-*test was used to compare two groups (*vs* Cas9 protein). The *P*-values are indicated on the panel. (**e**) Representative plots of zebrafish behavior trajectories (red lines) at 7 days post-fertilization (dpf) or 4 days post-SCI after spi1 knockdown. (**f**) Left panel: total distance moved and speed of uninjured zebrafish larvae for 10 min at 7 dpf following spi1 mutation, *n* = 30 zebrafish per group. Right panel: total distance moved and speed of injured zebrafish (SCI 4 d) larvae for 10 min at 7 dpf following spi1 mutation, *n* = 20 zebrafish per group. Data are presented as mean ± SD. Unpaired Student's *t-*test was used to compare two groups (*vs* ctrl). The *P*-values are indicated on the panel. (**g**) Left panel: representative sequential images showing swimming movement over a 10-second period (0, 2, 4, 6, 8, 10 s) in Cas9 protein control and Cas9 protein + spi1a/1b sgRNA groups following SCI 4d. Right panel: quantification of the maximum body-tail angle (rad) during the escape response. Data are presented as mean ± SD. Unpaired Student's *t-*test was used to compare two groups (*vs* Cas9 protein). The *P*-values are indicated on the panel. *SCI* spinal cord injury

**Figure 4:**
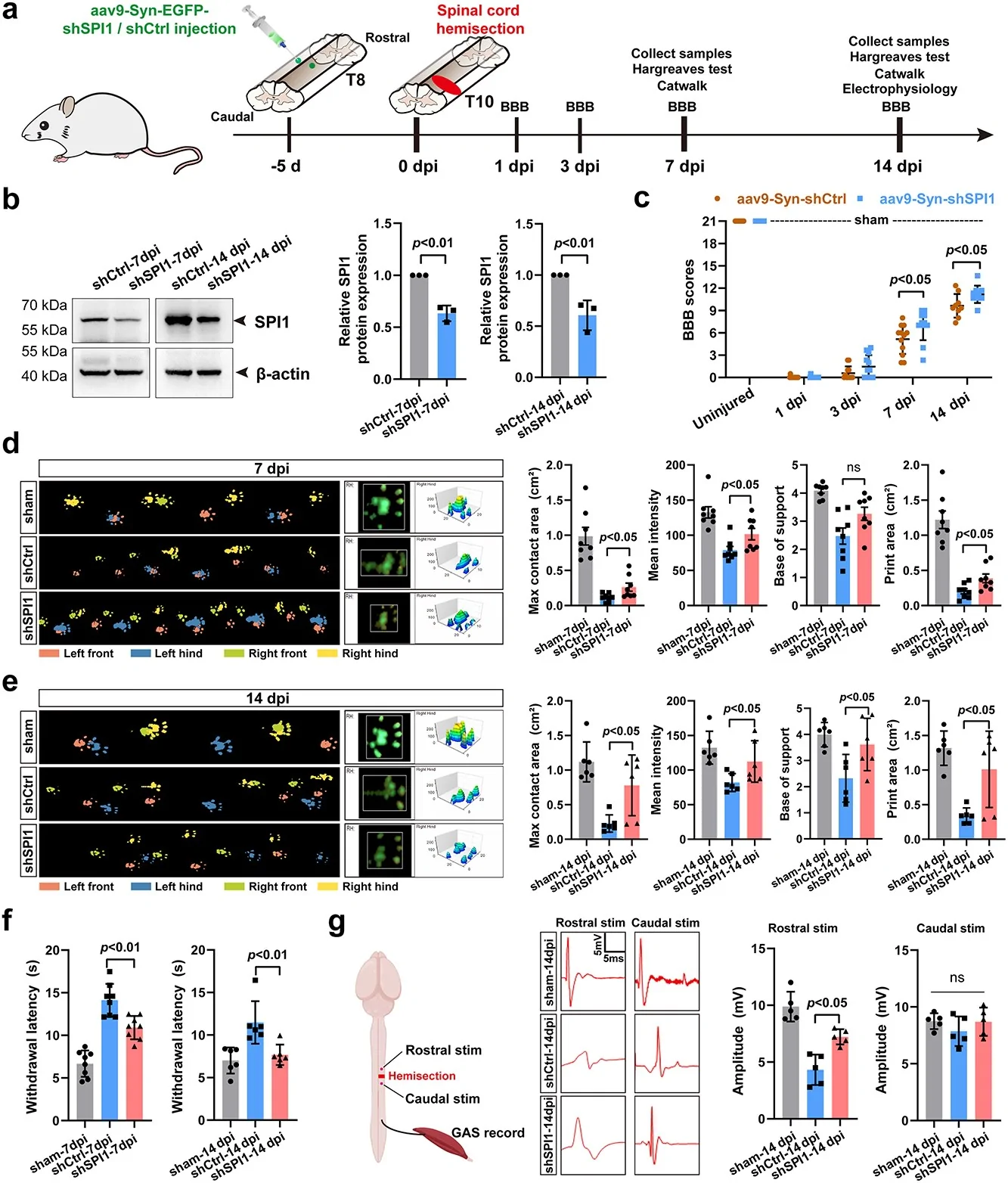
SPI1 knockdown in rat neurons facilitates functional recovery post-SCI (**a**) schematic of the experimental timeline for spinal cord hemisection and AAV9-Syn-EGFP-shCtrl or shSPI1 viral injection. Injections were performed at the thoracic level 8 (T8), followed by hemisection at T10. The timeline indicates sample collection and behavioral testing post-surgery. (**b**) SPI1 knockdown efficiency analysis. Left panel: representative western blot images showing SPI1 protein levels in spinal cord tissue at 7 and 14 days post-injury (dpi) following hemisection. Right panel: quantification of relative SPI1 protein expression normalized to β-actin. Data are presented as mean ± SD, *n* = 3 biological replicates. Unpaired Student's *t-*test was used to compare two groups (*vs* shCtrl). The *P*-values are shown on the panel. (**c**) BBB scores for rats treated with AAV9-Syn-EGFP-shCtrl or shSPI1 at various time points post-injury. Data are presented as mean ± SD, *n* = 9–12 rats per group. Statistical significance was determined using two-way ANOVA followed by Bonferroni's post hoc test (*P* < 0.05). The *P*-values are shown on the panel. (**d**) Catwalk gait analysis at 7 dpi following spinal cord hemisection. Left panel: representative sequences of footprints captured by the Catwalk system for sham, shCtrl-7dpi, and shSPI1-7dpi groups. Right panel: quantification of gait parameters, including maximum contact area (cm^2^), mean intensity (foot pressure), base of support (distance between lateral footprints), and print area (cm^2^). Data are presented as mean ± SD, with *n* = 8 rats per group. Statistical significance was assessed using unpaired Student's *t-*test; *P*-values are shown on the panel. (**e**) Catwalk gait analysis at 14 dpi following spinal cord hemisection. Left panel: representative sequences of footprints for sham, shCtrl-14dpi, and shSPI1-14dpi groups. Right panel: summary of key gait parameters: maximum contact area, mean intensity, base of support, and print area. Data are presented as mean ± SD, with *n* = 6 rats per group. Statistical significance was assessed using unpaired *t*-tests; *P*-values are shown on the panel. (**f**) PWL (seconds) as a measure of thermal pain threshold for rats treated with shCtrl and shSPI1 at 7 dpi (left panel, *n* = 8 rats per group) and 14 dpi (right panel, *n* = 6 rats per group). Data are presented as mean ± SD. Unpaired Student's *t-*test was used to compare two groups (*vs* shCtrl). The *P*-values are shown on the panel. (**g**) Electrophysiological assessment of spinal cord function at 14 dpi. Left panel: schematic of experimental setup for rostral and caudal stimulation, with GAS muscle recording. Middle panel: representative traces of evoked potentials recorded from the GAS muscle following rostral and caudal stimulation in sham-14dpi, shCtrl-14dpi, and shSPI1-14dpi groups. Right panel: quantification of the amplitude of evoked potentials following rostral (left) and caudal (right) stimulation. Data are presented as mean ± SD, with *n* = 5 rats per group. Statistical significance was assessed using one-way ANOVA; *P*-values are shown on the panel

We next assessed axonal outgrowth in spi1-disrupted crispant embryos. The lengths of eGFP-labeled motor-neuron axons were imaged and quantified at 30 hpf. As shown in Figure 3b and c, spi1 mutation led to a significant increase in axon length compared with the controls. These results are consistent with the phenotype observed in rat E14 spinal cord neurons following SPI1 knockdown, suggesting a conserved role for SPI1 in the regulation of axon growth across species.

Next, the impact of spi1 mutation on injured spinal cord repair was explored. Zebrafish were subjected to SCI at 3 days post-fertilization (dpf), and the number of axons crossing the lesion site was assessed at 1 day post-SCI (d/SCI). As depicted in Figure 3d, significantly more axons crossed the injury site in zebrafish with spi1 mutation than in control zebrafish.

Motor behavioral assays were performed in zebrafish at 4 d/SCI to evaluate functional recovery following axon regeneration. These assays measured the total swimming distance and velocity over a 10-min period. As shown in Figure 3e and f, compared with control zebrafish, zebrafish with spi1 mutation demonstrated greater total swimming distance and higher movement velocity (*P* <0.05), indicating improved motor function after SCI.

Finally, body–tail kinematics during swimming were analyzed to further assess the recovery of locomotor behavior. Representative sequential images of body and tail movements at 4 d/SCI are shown in Figure 3g. The quantitative analysis revealed a significant increase in the maximum body–tail angle in the spi1 mutation group compared with that in the control group (*P* < 0.05; Fig. 3g ), highlighting enhanced locomotor performance following SCI.

### Neuronal-specific knockdown of SPI1 enhances functional recovery and axon regeneration after spinal cord hemisection in rats

A neuron-specific AAV vector encoding shRNA against SPI1 (syn–EGFP–shSPI1) was developed to selectively knockdown SPI1 in neurons and investigate the specific role of SPI1 in axon regeneration. The neuron specificity of the synapsin promoter-driven AAV system was validated *in vivo*. IF staining of spinal cord sections revealed that AAV–syn–GFP predominantly colocalized with the neuronal marker NeuN but not with the astrocytic marker GFAP or the microglial marker IBA1 (Supplementary Fig. S2a and b). The knockdown efficiency of syn–EGFP–shSPI1 was further validated in cultured E14 spinal neurons. At 7 days post-transduction, Spi1 mRNA levels were significantly reduced in cells transduced with syn–EGFP–shSPI1 compared to cells transduced with the control shRNA vector (Supplementary Fig. S2c), confirming the specificity and efficacy of the knockdown method. Following the establishment of the spinal cord hemisection model, the transcriptional profiling data from our previous studies on spinal cord hemisection were reviewed [35]. Consistent with these findings, the results of the qPCR assay confirmed that SPI1 expression was significantly upregulated after SCI (Supplementary Fig. S2d and e).

AAV was administered at the T8 level of the spinal cord five days prior to injury, ensuring efficient transduction and shRNA expression. Spinal cord hemisection was then performed, and behavioral tests were conducted at various post-injury time points (dpi) to assess functional recovery (Fig. 4a). The results of the WB analysis showed a significant reduction in SPI1 protein expression in the shSPI1 groups compared with that in the control (shCtrl) groups at both 7 dpi and 14 dpi (Fig. 4b), confirming the successful knockdown of SPI1 *in vivo*. Behavioral assessments, including the BBB locomotor rating scale, Hargreaves test, and CatWalk analysis, were conducted at 7 dpi and 14 dpi. The shSPI1 group displayed significantly improved BBB scores, reduced thermal hyperalgesia (indicated by lower Hargreaves test scores), and better gait parameters according to the CatWalk analysis compared with the shCtrl group, suggesting that SPI1 knockdown promotes functional recovery after SCI (Fig. 4c–f). Electrophysiological assessments were conducted at 14 dpi to evaluate spinal cord conduction function. Representative traces of evoked potentials recorded from the GAS muscle following rostral and caudal stimulation at the injury site showed that the shSPI1 group exhibited a significantly increased amplitude of evoked potentials upon rostral stimulation compared with the shCtrl group, although no significant difference was observed following caudal stimulation (Fig. 4g). These findings suggest that SPI1 knockdown enhances neural signal transmission across the injury site, thereby further supporting its role in promoting functional recovery after SCI.

IF staining revealed that the GFP fluorescence intensity was significantly higher at the injury site in the shSPI1 group than in the shCtrl group at both 7 and 14 dpi. Similarly, the fluorescence intensity of GAP43, a marker of regenerating axons, was markedly increased in the shSPI1 group at both time points (Fig. 5a–d). Notably, GFP and GAP43 partially colocalized in the shSPI1 group, with GFP/GAP43 double-positive cells clearly visible (arrowheads in Fig. 5a and b). A line profile analysis across the injury site further confirmed the overlapping distribution patterns of GFP and GAP43 fluorescence at both 7 and 14 dpi in the shSPI1 group (Fig. 5e), suggesting that GFP-positive transduced neurons were closely associated with GAP43-positive regenerating axons.

NF200-positive axonal fibers and serotonin (5-HT)-positive descending fibers at the injury site were examined at 14 dpi to further characterize the regenerative response. Both the NF200 and 5-HT fluorescence intensities were significantly higher in the shSPI1 group than in the control group (Fig. 5f and g). At higher magnification, GFP colocalized with NF200-positive fibers (white arrowheads; Fig. 5f) and 5-HT-positive fibers (yellow arrows; Fig. 5f), indicating that SPI1 knockdown promotes general axonal growth and facilitates the regeneration of descending serotonergic projections within the transduced region.

These results demonstrate that SPI1 knockdown enhances axonal regeneration at the lesion site following SCI, highlighting its potential as a therapeutic target for promoting neural repair and functional recovery.

### SPI1 transcriptionally activates Rassf10 to modulate axonal growth

Key downstream effectors were identified to elucidate the transcriptional cascade underlying the SPI1-mediated regulation of axonal growth. Initially, RNA-sequencing datasets obtained by our research group at E18, P1, and P3 [34] were analyzed to screen for genes whose expression patterns were consistent with those of SPI1. The Cut&Tag-seq technique was subsequently employed to enrich for potential SPI1-binding target genes (i.e. genes that were upregulated in the SPI1 group relative to the IgG control group). RNA sequencing was also performed to identify genes whose expression was downregulated following SPI1 silencing via an siRNA. A total of 14 genes were identified from the intersection of these three gene sets, including Rassf10, Ust, Slc15a3, Gbp4, Fam241a, Tnfrsf1b, Pld4, Adgre1, Anxa3, Hcls1, Mx1, Cyp4v3, Nlrc5, and Hvcn1 (Fig. 6a and b and Supplementary Table S2). A GO enrichment analysis was conducted on the Cut&Tag-seq data, focusing on biological processes (BPs) related to the nervous system (Fig. 6b and Supplementary Table S2). This analysis highlighted Rassf10 and Tnfrsf1b as candidate genes, with Rassf10 selected for further investigation because of its unexplored role in axonal biology, in contrast to the previously reported association between Tnfrsf1b and axonal function [36].

The initial analysis of sequencing data from the E18, P1, and P3 stages revealed a positive correlation between Rassf10 and SPI1 expression (Supplementary Fig. S1d). *In vivo* studies demonstrated that SPI1 knockdown led to a significant reduction in Rassf10 expression (Fig. 6c). Further manipulation of SPI1 expression in primary cultured neurons showed that SPI1 knockdown resulted in a marked decrease in Rassf10 expression, consistent with the *in vivo* findings, whereas SPI1 overexpression significantly upregulated Rassf10 expression (Fig. 6d). These results confirm that SPI1 positively regulates Rassf10 expression. Moreover, the genetic manipulation of neuronal Rassf10 expression recapitulated the axonal phenotypes observed with SPI1 perturbation. As shown in Figure 6e–h, Rassf10 silencing markedly increased the length of axons: the average axonal length of the siRassf10-treated neurons reached 74.53 ± 4.534 μm, representing an 18.5% increase compared with that of the siCtrl-treated neurons (62.88 ± 2.653 μm). Conversely, ectopic Rassf10 expression strongly suppressed axon outgrowth, with the mean axonal length (53.60 ± 2.203 μm) of neurons transfected with pcDNA3.1–Rassf10 being 20.5% shorter than that of the control neurons transfected with the empty vector (67.44 ± 3.073 μm).

**Figure 5:**
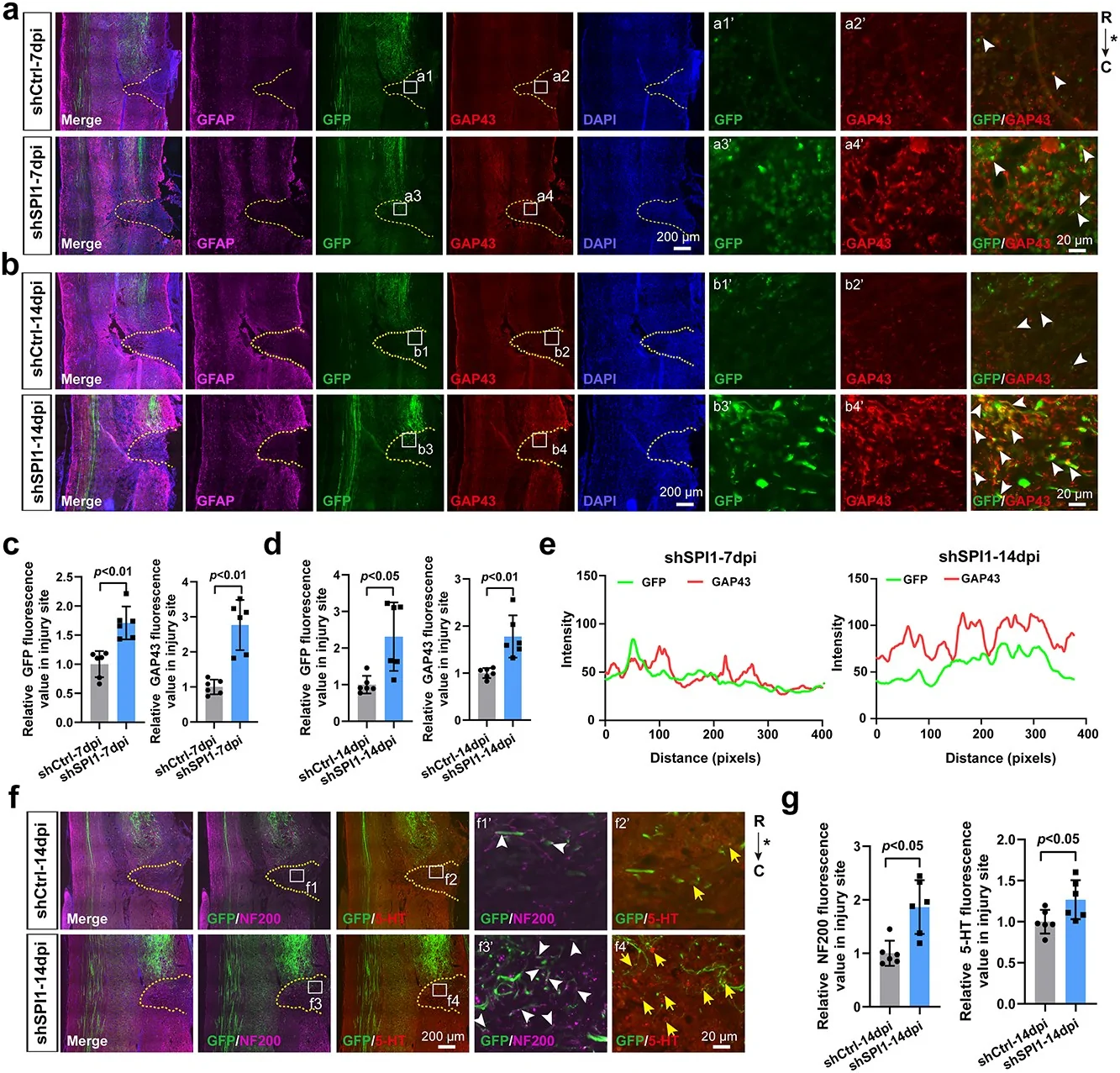
SPI1 knockdown in rat neurons facilitates axon growth post-SCI (**a**) representative images of GFAP, GFP, GAP43, and DAPI staining at 7 dpi. GFAP expression marks the injury margins, with the distribution of GAP43-positive and GFP-positive cells at the injury site. The dotted lines delineate the injury area. Panels a1–a4 and a1′–a4′ show magnified views of the boxed regions. Arrowheads highlight GFP/GAP43 double-positive cells. R, rostral; C, caudal; asterisks indicate the injured side. Scale bar: 200 μm (low magnification), 20 μm (high magnification). (**b**) Representative images of GFAP, GFP, GAP43, and DAPI staining at 14 dpi. GFAP expression marks the injury margins, and GAP43 and GFP distributions are shown at the injury site. The dotted lines delineate the injury area. Panels b1–b4 and b1′–b4′ show magnified views of the boxed regions. Arrowheads highlight GFP/GAP43 double-positive cells. Scale ba: 200 μm (low magnification), 20 μm (high magnification). (**c**) Statistical analysis of relative GFP and GAP43 fluorescence intensity at the injury site at 7 dpi. Data are presented as mean ± SD, *n* = 6 rats per group. Unpaired Student's *t-*test was used to compare two groups (*vs* shCtrl). The *P*-values are shown on the panel. (**d**) Statistical analysis of relative GFP and GAP43 fluorescence intensity at the injury site at 14 dpi. Data are presented as mean ± SD, *n* = 6 rats per group. Unpaired Student's *t-*test was used to compare two groups (*vs* shCtrl). The *P*-values are shown on the panel. (**e**) Fluorescence intensity line profiles of GFP and GAP43 across the injury site at 7 dpi and 14 dpi in the shSPI1 group. (**f**) Representative images of GFP, NF200, 5-HT, and DAPI (merge) staining at 14 dpi. Distribution of NF200-positive fibers and 5-HT-positive fibers at the injury site. The dotted lines outline the injury area. Panels f1–f4 and f1′–f4′ show magnified views of the boxed regions. White arrowheads highlight GFP/NF200 double-positive fibers, and yellow arrowheads highlight GFP/5-HT double-positive fibers. R, rostral; C, caudal; asterisks indicate the injured side. Scale bar: 200 μm (low magnification), 20 μm (high magnification). (**g**) Statistical analysis of relative NF200 and 5-HT fluorescence intensity at the injury site at 14 dpi. Data are presented as mean ± SD, *n* = 6 rats per group. Unpaired Student's *t-*test was used to compare two groups (*vs* shCtrl). The *P*-values are shown on the panel

**Figure 6:**
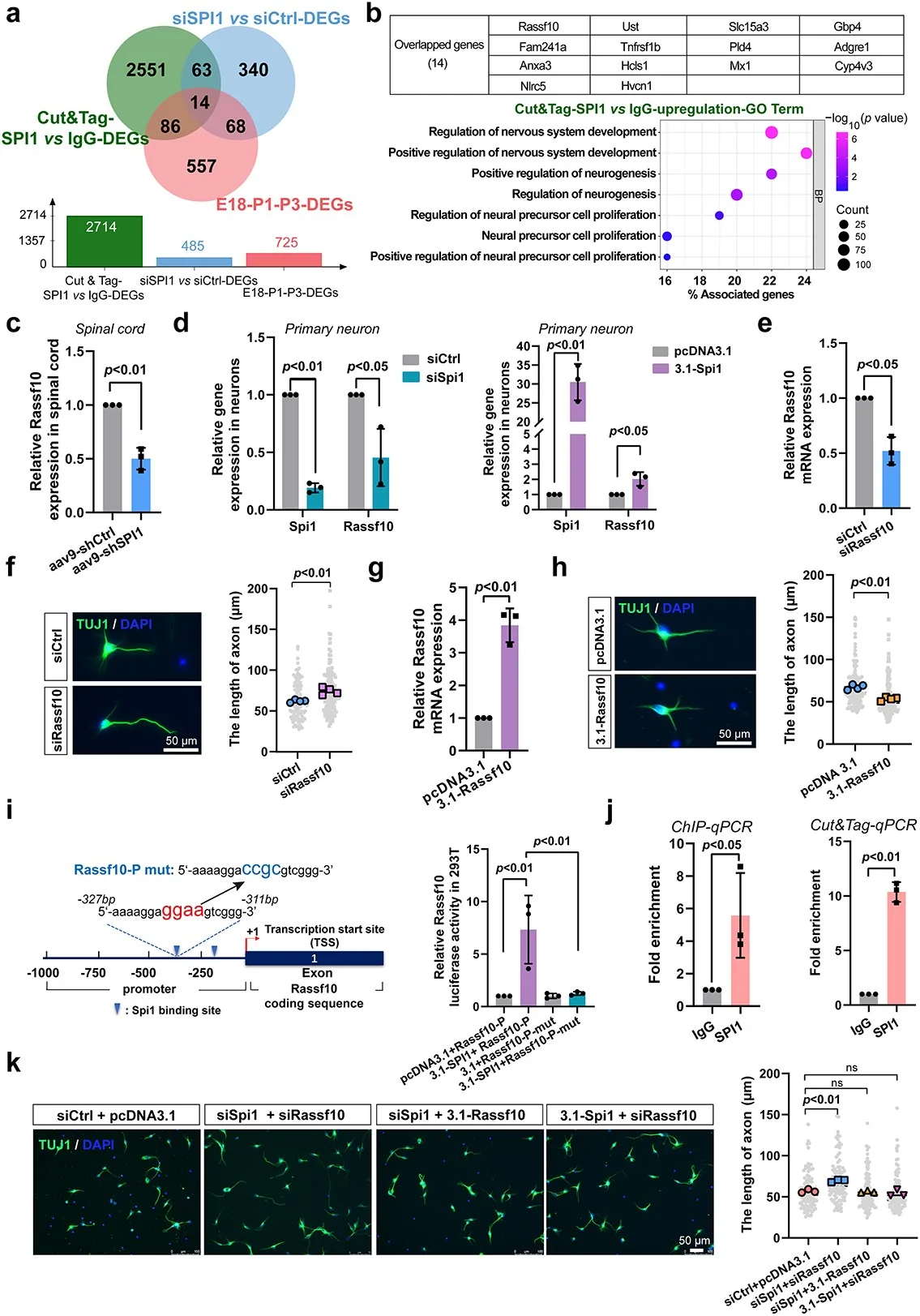
SPI1 regulates axon outgrowth through Rassf10 (**a**) Venn diagram showing the overlap of DEGs from three comparison groups: siSPI1 *vs* siCtrl-DEGs (downregulation genes, knockdown of SPI1 *vs* control), Cut&Tag-SPI1 *vs* IgG-DEGs (upregulation genes, SPI1 chromatin binding *vs* IgG control), and E18-P1-P3-DEGs (upregulation genes, developmental stage comparison: E18 *vs* P1 *vs* P3). The bar chart below quantifies the number of unique DEGs for each comparison. (**b**) Up panel: table listing the 14 overlapping genes identified across the three comparison groups. Lower panel: dot plot showing GO enrichment analysis of genes upregulated in the cut&tag-SPI1 *vs* IgG comparison, with a focus on nervous system-related biological processes. (**c**) qPCR detection of Rassf10 expression after aav9-syn-EGFP-shSPI1 or shCtrl treatment in rats at 21 d. Data are presented as mean ± SD, *n =* 3 biological replicates. Unpaired Student's *t-*test was used to compare two groups (*vs* shCtrl). The *P*-values are shown on the panel. (**d**) Left panel: the expression of Rassf10 after SPI1 siRNA treatment in neurons. Right panel: the expression of Rassf10 after SPI1 overexpression treatment in neurons. Data are presented as mean ± SD, *n =* 3 biological replicates. Unpaired Student's *t-*test was used to compare two groups (*vs* siCtrl/pcDNA3.1). The *P*-values are shown on the panel. (**e**) qPCR detection of Rassf10 knockdown efficiency in neurons. Data are presented as mean ± SD, *n =* 3 biological replicates. Unpaired Student's *t-*test was used to compare two groups (*vs* siCtrl). The *P*-values are shown on the panel. (**f**) Effects of Rassf10 siRNA on axonal growth. Left panel: the representative IF images stained with Tuj1 antibody with a scale bar of 50 μm. Right panel: statistical analysis. Data are presented as mean ± SD, *n =* 4 biological replicates, the gray dots represent 120 neurons. Unpaired Student's *t-*test was used to compare two groups (*vs* siCtrl). The *P*-values are shown on the panel. (**g**) qPCR detection of Rassf10 overexpression efficiency in neurons. Data are presented as mean ± SD, *n =* 3 biological replicates. Unpaired Student's *t-*test was used to compare two groups (*vs* pcDNA3.1). The *P*-values are shown on the panel. (**h**) Effects of Rassf10 overexpression on axonal growth. Left panel: the representative IF images stained with Tuj1 antibody with a scale bar of 50 μm. Right panel: statistical analysis. Data are presented as mean ± SD, *n =* 4 biological replicates, the gray dots represent 120 neurons. Unpaired Student's *t-*test was used to compare two groups (*vs* siCtrl). The *P*-values are shown on the panel. (**i**) Left panel: diagrammatic sketch of the possible binding sites of the SPI1 transcription factor predicted within −1000 bp to +1 bp of the Rassf10 gene promoter. Right panel: results of luciferase reporter analysis. (**j**) Left panel: representative results of ChIP-qPCR analysis. Right panel: representative results of Cut&taq-qPCR analysis. The anti-IgG/SPI1 antibody immunoprecipitated the IgG/SPI1 -DNA complex; the DNA region bound by IgG/SPI1 was identified by real-time quantitative PCR (qPCR) using Rassf10-specific primers, IgG was used as immunoprecipitated controls. Data are presented as mean ± SD, *n =* 3 biological replicates. Unpaired Student's *t-*test was used to compare two groups (*vs* IgG). (**k**) Effects of siCtrl+pcDNA3.1, siSpi1 + siRassf10, siSPI1 + 3.1-Rassf10, and 3.1-SPI1 + siRassf10 treatment on axonal growth. Left panel: the representative IF images stained with Tuj1 antibody with a scale bar of 50 μm. Right panel: statistical analysis. Data are presented as mean ± SD, *n =* 3 biological replicates, the gray dots represent 90 neurons. Unpaired Student's *t-*test was used to compare two groups (*vs* siCtrl+pcDNA3.1). “Ns” stands for no significance

Given the regulatory effect of SPI1 on Rassf10 and their critical roles in modulating neuronal axonal growth, we next investigated the molecular mechanism by which SPI1 governs Rassf10 transcription. A panel of transcription factor prediction databases was consulted to identify the upstream regulators of Rassf10, and SPI1 was identified as a key transcription factor for Rassf10 (Supplementary Fig. S3a). The JASPAR and AnimalTFDB (v4.0) databases were subsequently used to predict the binding region of SPI1 within the Rassf10 promoter (spanning −1000 bp to +1 bp), and luciferase reporter assays were conducted to validate the regulatory relationship between SPI1 and Rassf10. An analysis of the sequence revealed a canonical SPI1 binding motif (5′aaaaggaGGAAgtcggg3′) located at positions −327 to −311 bp upstream of the Rassf10 transcription start site (TSS). This motif was disrupted via site-directed mutagenesis to generate a mutant reporter construct, Rassf10-P mut (5′aaaaggaCCGCgtcggg3′). Dual-luciferase assays performed in 293 T cells showed that SPI1 overexpression substantially increased wild-type Rassf10 promoter activity (*P* < 0.01), whereas the mutation of this motif largely abolished SPI1-dependent transactivation (Fig. 6i). These results indicated that the identified cis-element is essential for SPI1-mediated transcription. We further validated endogenous binding of SPI1 to the Rassf10 promoter in neurons by performing ChIP assays. The qPCR analysis of immunoprecipitated DNA using target-specific primers revealed a 5.58-fold enrichment of the Rassf10 promoter in the SPI1 pulldown group relative to that in the IgG control group (*P* < 0.05; Fig. 6j, left panel**)**, and this prominent enrichment in the SPI1 group was further confirmed by conventional PCR followed by electrophoresis (Supplementary Fig. S3b). qPCR amplification with nontargeting binding primers still yielded a 2.15-fold enrichment in the SPI1 group (ns, Supplementary Fig. S3c). We performed Cut&Tag–qPCR to further verify this binding interaction with reduced background noise, and the results showed that the enrichment efficiency of the SPI1 group was increased by 10.36-fold compared with that of the IgG control group (*P* < 0.01; Fig. 6j, right panel). Together, these results demonstrate that SPI1 directly binds to the −327 to −311 bp motif within the Rassf10 proximal promoter to induce its expression.

Finally, rescue experiments were conducted to confirm the relationship between SPI1 and Rassf10. TUJ1/DAPI costaining was performed to visualize axonal morphology in neurons subjected to four combinatorial transfections: siCtrl + pcDNA3.1, siSpi1 + siRassf10, siSpi1 + Rassf10 overexpression plasmid, and SPI1 overexpression plasmid + siRassf10. The quantification of the axonal length revealed that the dual knockdown of Spi1 and Rassf10 significantly increased the axonal length compared with control neurons, whereas Spi1 knockdown combined with Rassf10 overexpression, as well as Spi1 overexpression combined with Rassf10 knockdown, restored the axonal length to the control levels (Fig. 6k). These results demonstrate that SPI1 regulates axonal length at least in part through its downstream target gene *Rassf10*.

### The p38 MAPK signaling pathway is involved in axon outgrowth regulated by the SPI1–Rassf10 axis

We explored the downstream signaling pathways regulated by the SPI1–Rassf10 axis in controlling axonal growth by performing transcriptome sequencing on neurons with SPI1 knockdown. GO and KEGG analyses of siSPI1-induced DEGs revealed that the downregulated genes were enriched mainly in immune and inflammatory pathways, including “immune system process”, “inflammatory response”, “cytokine–cytokine receptor interaction” and “NF-κB signaling pathway” (Supplementary Fig. S4a and b and Table S1). Moreover, the upregulated DEGs were enriched in distinct functional categories, with “cell surface”, “angiotensin receptor activity” and “cellular extravasation” as the top terms in the cellular component, molecular function, and biological process categories, respectively. Notably, the KEGG pathway enrichment analysis of upregulated DEGs revealed the significant enrichment of the MAPK signaling pathway, which contained the greatest number of enriched genes (Fig. 7a and b and Supplementary Table S1).

**Figure 7:**
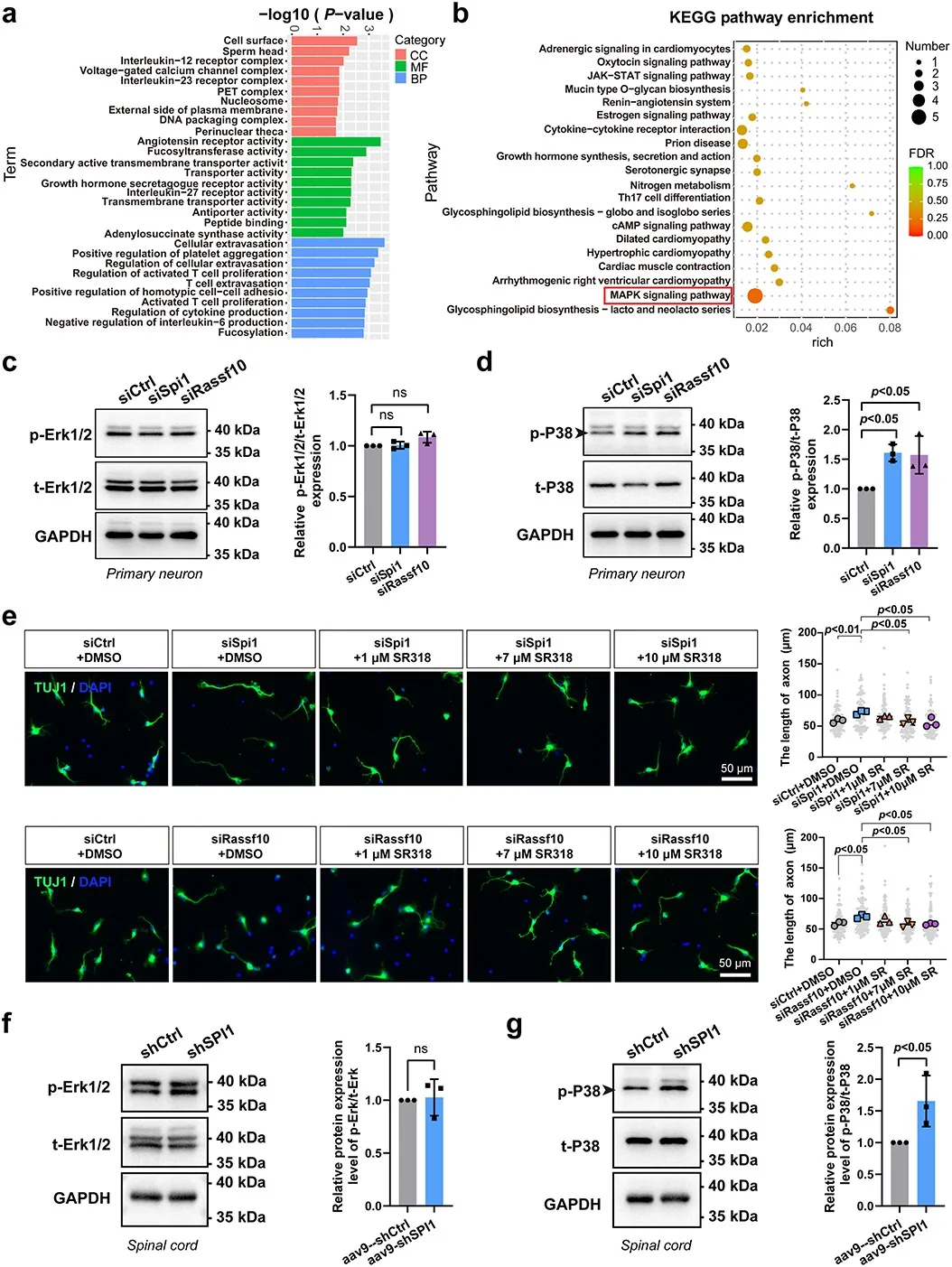
p38 MAPK signaling pathway is involved in SPI1-Rassf10 axis-regulated axon outgrowth (**a**) GO terms from the GO enrichment analysis of upregulated genes following siSPI1 treatment in neurons. (**b**) KEGG analysis of upregulated genes after siSPI1 treatment in neurons. (**c**) Protein expression of p-Erk1/2 and total Erk1/2 following Spi1/Rassf10 siRNA treatment in neurons. Left panel: representative western blot results; right panel: statistical analysis. Data are presented as mean ± SD, *n* = 3 biological replicates. Unpaired Student's *t-*test was used to compare two groups (*vs* siCtrl). “Ns” indicates no significant difference. (**d**) Protein expression of p-P38 MAPK and total P38 MAPK after Spi1/Rassf10 siRNA treatment in neurons. Left panel: representative western blot results; right panel: statistical analysis. Data are presented as mean ± SD, *n* = 3 biological replicates. Unpaired Student's *t-*test was used to compare two groups (*vs* siCtrl). The *P*-values are shown on the panel. (**e**) Impact of SR318 (p-P38 MAPK specific inhibitor) on axonal growth after Spi1/Rassf10 siRNA treatment. Left panel: representative IF images of Tuj1 treated with SR318 (1, 7, and 10 μM) for 48 h in neurons. Scale bar: 50 μm. Right panel: statistical analysis. Data are presented as mean ± SD, *n* = 3 biological replicates. The gray dots represent 90 neurons. Unpaired Student's *t-*test was used to compare two groups (*vs* siCtrl + DMSO). The *P*-values are shown on the panel. (**f**) Protein expression of p-Erk1/2 and total Erk1/2 after aav9-syn-EGFP-shSPI1/shCtrl treatment in rats at 7 dpi. Left panel: representative western blot results; right panel: statistical analysis. Data are presented as mean ± SD, *n* = 3 biological replicates. Unpaired Student's *t-*test was used to compare two groups (*vs* shCtrl). “Ns” indicates no significant difference. (**g**) Protein expression of p-P38 MAPK and total P38 MAPK after aav9-syn-EGFP-shSPI1/shCtrl treatment in rats at 7 dpi. Left panel: representative western blot results; right panel: statistical analysis. Data are presented as mean ± SD, *n* = 3 biological replicates. Unpaired Student's *t-*test was used to compare two groups (*vs* shCtrl). The *P*-values are shown on the panel

We next examined whether SPI1 and Rassf10 affect the MAPK signaling pathway. Protein lysates were collected from neurons 48 h after SPI1 or Rassf10 knockdown for WB analysis. An initial examination revealed that Erk1/2 phosphorylation did not change significantly following SPI1 or Rassf10 knockdown (Fig. 7c). Previous studies have suggested that the activation of the p38 MAPK pathway promotes axonal growth [14–16], whereas Rassf10 has been shown to inhibit p38 MAPK signaling [37]. We further assessed whether SPI1 or Rassf10 knockdown affects p38 MAPK signaling and found that the depletion of either gene significantly increased p38 MAPK phosphorylation by >1.5-fold (*P* < 0.05; Fig. 7d). We tested whether suppressing this increase in p38 MAPK phosphorylation could counteract the axonal phenotype by treating Spi1- or Rassf10-depleted neurons with PD169316, a p38 MAPK inhibitor. At 10 μM, PD169316 significantly attenuated the increased axonal outgrowth induced by Spi1 or Rassf10 knockdown (Supplementary Fig. S4c and d). Furthermore, we employed SR318, a more selective p38 MAPK inhibitor. Consistent with the results obtained using PD169316, both 7 μM and 10 μM SR318 significantly suppressed the increase in axonal outgrowth in Spi1- or Rassf10-depleted neurons (Fig. 7e). Together, these results indicate that p38 MAPK signaling acts downstream of the SPI1–Rassf10 axis to regulate axonal growth.

Finally, we validated the regulatory effect of SPI1 on MAPK signaling *in vivo* using neuron-specific SPI1 silencing in the spinal cord. Consistent with our *in vitro* results, WB analysis of spinal cord tissues showed that neuronal SPI1 knockdown significantly increased p38 MAPK phosphorylation relative to the level of the total p38 MAPK protein (*P* < 0.05; Fig. 7g). In contrast, the ratio of phosphorylated to total Erk1/2 remained unaltered between the control and SPI1-silenced groups (Fig. 7f), indicating that SPI1 deletion selectively activated the p38 MAPK cascade without affecting Erk signaling.

In summary, we identified SPI1 as a transcriptional activator of Rassf10 that directly binds to its promoter in neurons. The depletion of either SPI1 or Rassf10 promotes axonal outgrowth and functional recovery, and this effect is mediated by increased p38 MAPK phosphorylation (Fig. 8).

**Figure 8:**
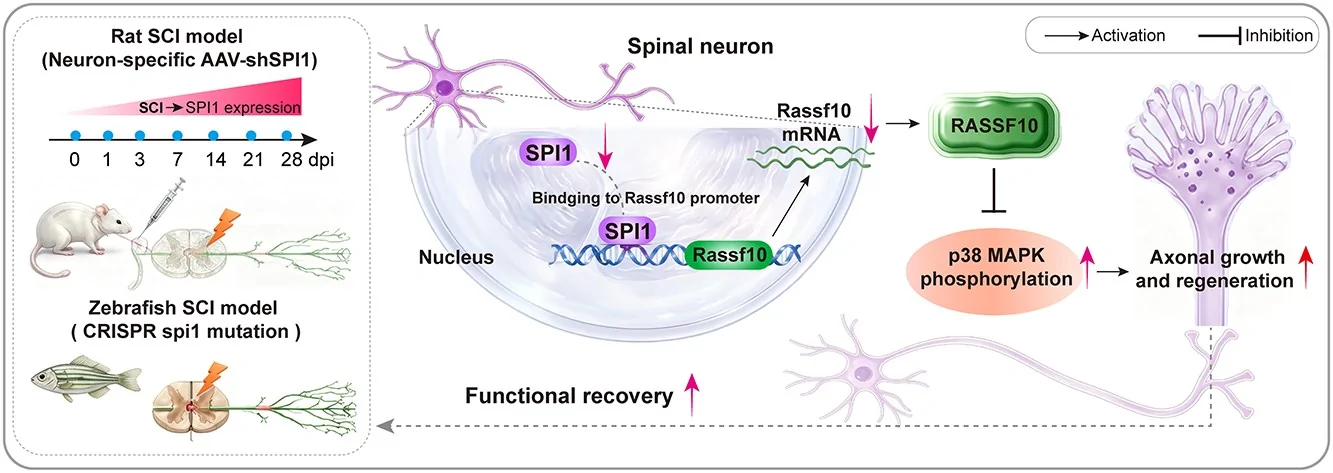
Schematic representation of the role of SPI1 in axonal regeneration following SCI. Following SCI, SPI1 expression is dynamically upregulated from 0 to 28 days post-injury. Downregulation of SPI1 expression significantly enhances functional recovery in both rats and zebrafish after SCI. SPI1 directly regulates the expression of Rassf10 in neurons, which in turn modulates the p38 MAPK signaling pathway. The diagram illustrates the proposed molecular mechanism by which SPI1 negatively regulates axonogenesis through the Rassf10 and p38 MAPK axis, highlighting the therapeutic potential of targeting this pathway to enhance neural repair. (Original image materials for this figure were generated by GPT image 2 model and Doubao, and further edited with Adobe Illustrator 2025.) *SCI* spinal cord injury

## Discussion

This study fundamentally redefines SPI1 (PU.1) as a dual-function transcriptional regulator that operates beyond its classical immunological role. While traditionally recognized as a master regulator of myeloid and immune cell fate, SPI1 also functions as an intrinsic suppressor of axon growth in neurons, as shown in the present study. Axonal regeneration remains essential for functional recovery following CNS injury. Previous research has demonstrated that both intrinsic and extrinsic factors, such as increased mTOR activity and neuronal electrical activity, can promote axonal regeneration [38, 39]. Additionally, strategies such as neural stem cell transplantation or the overexpression of specific transcription factors have been shown to reprogram mature neurons to a developmental-like state, facilitating axonal growth and recovery [40]. Moreover, the transcription factor c-Jun plays a pivotal role in promoting axonal growth, as its overexpression is sufficient to stimulate axonal elongation without additional modifications of gene expression [5, 41]. Despite these advances, the transcriptional networks that restrict axonal regeneration in neurons remain largely unexplored, representing a critical gap in neuroregeneration research.

While SPI1 is well established as a lineage-determining transcription factor for microglia and myeloid cells, emerging evidence suggests that SPI1 has nonimmune functions in the nervous system [8, 9, 42]. However, previous studies have focused primarily on microglia and macrophages, with no investigations into the role of SPI1 in axon biology. This study reveals a novel regulatory function for SPI1 in axon outgrowth through its downstream target gene Rassf10 and the p38 MAPK signaling pathway, providing new insights for neurobiology and regenerative medicine. Our data demonstrate that SPI1 expression is dynamically regulated during spinal cord development, with its neuronal expression gradually decreasing and a shift in its subcellular localization observed, which is correlated with a decrease in regenerative potential. This pattern suggests that SPI1 plays a critical role in early neuronal development and maturation. Notably, SCI triggers the marked upregulation of neuronal SPI1 expression, coupled with the increased nuclear translocation of this protein. Consistently, the genetic perturbation of SPI1 expression *in vitro* and *in vivo* profoundly altered axonal extension, corroborating that neuronal SPI1 represses axonal growth.

In zebrafish models, CRISPR/Cas9-mediated mutation of both spi1a and spi1b significantly promoted axon growth and injured spinal cord repair, as evidenced by the increased swimming velocity, total distance traveled, and maximum body–tail angles. This result may be due to spi1 mutation reducing the inflammatory response of microglia and macrophages [8], thereby improving the microenvironment for axon growth. Alternatively, the combined effects of these factors may also play a role. Neuron-specific silencing of SPI1 via AAV9 in rats further supports a cell-autonomous mechanism. In the spinal cord hemisection model, selectively reducing SPI1 expression in neurons significantly enhanced axonal regeneration and functional recovery, as reflected by improved BBB locomotor scores, enhanced gait kinematics, reduced thermal hyperalgesia, and increased numbers of GAP43^+^, NF200^+^, and 5-HT^+^ axonal fibers at the lesion site. These consistent findings across both mammalian and zebrafish models expand the understanding of the role of SPI1 beyond myeloid cells, highlighting its potential as a therapeutic target to promote axonal regeneration.

Transcription factors regulate biological processes by controlling the transcription of downstream target genes. This study demonstrated that Rassf10 is a downstream target of the transcription factor SPI1. Members of the RASSF family play critical roles in the neural repair process following injury. For example, RASSF7a promotes spinal cord regeneration and governs the asymmetric division of neural progenitor cells, thereby influencing neuronal regeneration after injury [43]. RASSF5 (NORE1) interacts with activated RAS in its GTP-bound state, inducing apoptosis [44], which may exert secondary protective effects on neurons or oligodendrocytes. Our findings show that SPI1 directly binds to the Rassf10 promoter, regulating its expression and consequently influencing axonal length. This relationship was confirmed by the results of luciferase reporter assays, ChIP, and Cut&Tag experiments, revealing a clear molecular mechanism by which SPI1 controls axon outgrowth. Rescue experiments further validated the functional relevance of the SPI1–Rassf10 axis in regulating axonal elongation. However, these results also indicate the involvement of additional target genes in mediating this effect, which will require further experimental investigation. A comprehensive study of the other 13 intersecting candidate genes, including Tnfrsf1b, Ust, and Slc15a3, through combined knockdown and epistasis analyses will be essential for fully mapping the SPI1-regulated transcriptional network controlling axonal plasticity.

The p38 MAPK signaling pathway is a well-established mediator of cellular stress responses and plays an essential role in neuronal survival and axon growth [45]. Transcriptome sequencing and subsequent bioinformatics analysis revealed the involvement of the MAPK signaling pathway in SPI1-regulated axon outgrowth. Specifically, p38 MAPK phosphorylation increased significantly after the separate knockdown of SPI1 or Rassf10 compared with the siCtrl group, supporting the hypothesis that the p328 MAPK pathway is modulated by SPI1 and Rassf10 to influence axonal growth. Notably, Rassf10 has been previously reported to negatively regulate p38 MAPK activity in non-neuronal contexts [37], and our study extends this observation to neurons, linking it directly to the control of axonal outgrowth. Given that the loss of SPI1 or Rassf10 increases p38 MAPK phosphorylation and drives excessive axonal elongation, we next tested whether blocking hyperactivated p38 MAPK signaling could reverse the axonal overgrowth phenotype. We treated Spi1- or Rassf10-depleted neurons with the p38 MAPK inhibitor PD169316 and found that 10 μM PD169316 effectively reduced the excessive axonal outgrowth triggered by the loss of SPI1 or Rassf10. However, published evidence indicates that PD169316 exhibits off-target activity at this dosage [46]. We replicated the rescue assays with SR318, a more selective p38 MAPK inhibitor, to exclude nonspecific confounding effects. We obtained identical functional outcomes: both 7 μM and 10 μM SR318 significantly restored normal axonal length in SPI1- and Rassf10-deficient neurons. The consistent rescue effects observed with both compounds confirm that p38 MAPK is the key downstream mediator linking the loss of SPI1/Rassf10 to altered axonal growth. In addition to the well-documented p38 MAPK pathway that regulates axonal growth, several other significantly enriched KEGG pathways coordinate to govern axonal outgrowth. The cAMP cascade enlarges growth cones by triggering PKA–CREB-mediated transcription of GAP43 and other regeneration-related genes [47]. Th17 cell differentiation modulates the inflammatory microenvironment, which in turn activates JAK–STAT3 signaling to drive the transcription of axon regeneration-associated genes [48]. Separately, altered glycosphingolipid biosynthesis reshapes the lipid composition of the axonal membrane and tunes cytoskeletal dynamics, thereby facilitating axonal extension and guidance [49]. Taken together, these interacting signaling cascades form a multilayered regulatory network that coordinates axonal repair after SCI. Future experiments will help clarify whether these pathways participate in SPI1–Rassf10-controlled axonal regulation.

Several limitations of the present study must be acknowledged. First, the spinal cord lateral hemisection model used here does not exclude the potential contribution of axonal sprouting from spared fibers, which may result in an overestimation of axonal regeneration. A spinal cord crush model will be utilized in future studies to more accurately assess genuine regeneration and better mimic clinical injury. Additionally, neuron-specific conditional SPI1 knockout mice will be generated to provide more definitive genetic evidence. Second, DEGs were identified based on unadjusted p values, which may increase the risk of false positives. Future RNA-seq analyses will incorporate more biological replicates and utilize adjusted p values (false discovery rate, FDR) for gene screening to increase the reliability of the transcriptomic data. Third, this study primarily adopted pharmacological inhibitors to verify the regulatory effect of the p38 MAPK pathway. We used two selective p38 inhibitors, SR318 and PD169316, to strengthen the reliability of our drug assays; however, small-molecule inhibitors commonly produce unintended off-target effects. Follow-up genetic studies, such as siRNA knockdown or gene knockout, are needed to confirm that the SPI1–Rassf10 axis controls axon growth/growth specifically through downstream p38 MAPK signaling.

Furthermore, reliably separating true axonal regeneration from local axonal sprouting remains technically challenging when standard staining and tracing assays are used, and these two phenomena lead to entirely different therapeutic development routes for SCI. Treatments designed to increase long-distance axonal regeneration focus on overcoming the intrinsic limits of neuronal growth and glial scar obstruction, whereas sprouting-targeted therapies work by rebuilding local neural circuits around the lesion for functional compensation. In our follow-up experiments, we will optimize the delivery of pathway inhibitors and confirm the *in vivo* efficacy of drugs targeting the SPI1–Rassf10 axis in multiple SCI animal models, with the ultimate goal of developing precise therapies that specifically induce genuine axonal regeneration.

## Conclusions

In summary, this study reveals a key regulatory mechanism involving SPI1, Rassf10, and the p38 MAPK pathway in axonal outgrowth, providing new insights into the intrinsic molecular mechanisms of neural regeneration after SCI. Cross-species verification in zebrafish and rat models confirms the translational value of our results, as CRISPR/Cas9 spi1 mutation in zebrafish and neuron-targeted AAV9–shRNA–mediated SPI1 silencing in rats increase axonal regrowth, sprouting and post-SCI functional restoration. Mechanistically, SPI1 directly binds to and transactivates the Rassf10 promoter, and elevated Rassf10 expression suppresses p38 MAPK phosphorylation to inhibit axonal elongation, collectively advancing the understanding of axonal growth regulation and identifying the SPI1–Rassf10 axis and p38 MAPK as promising therapeutic targets for SCI and other disorders characterized by impaired axonal regeneration.

## Supplementary Material

Supplementary_material_tkag054

## Data Availability

The data that support the findings of this study are available from the corresponding author upon reasonable request.
