# Supplementary material for: Neuronal SPI1 suppression enhances axonal regeneration after spinal cord injury through Rassf10 downregulation
Source: Burns Trauma. 2026 Aug 13;14:tkag054. doi: 10.1093/burnst/tkag054 (PMC13623519; doi:10.1093/burnst/tkag054)
Supplement: Supplementary file 1 [file supplementary_material_tkag054.zip › Supplementary_Figures-26.8.10_tkag054.pdf]

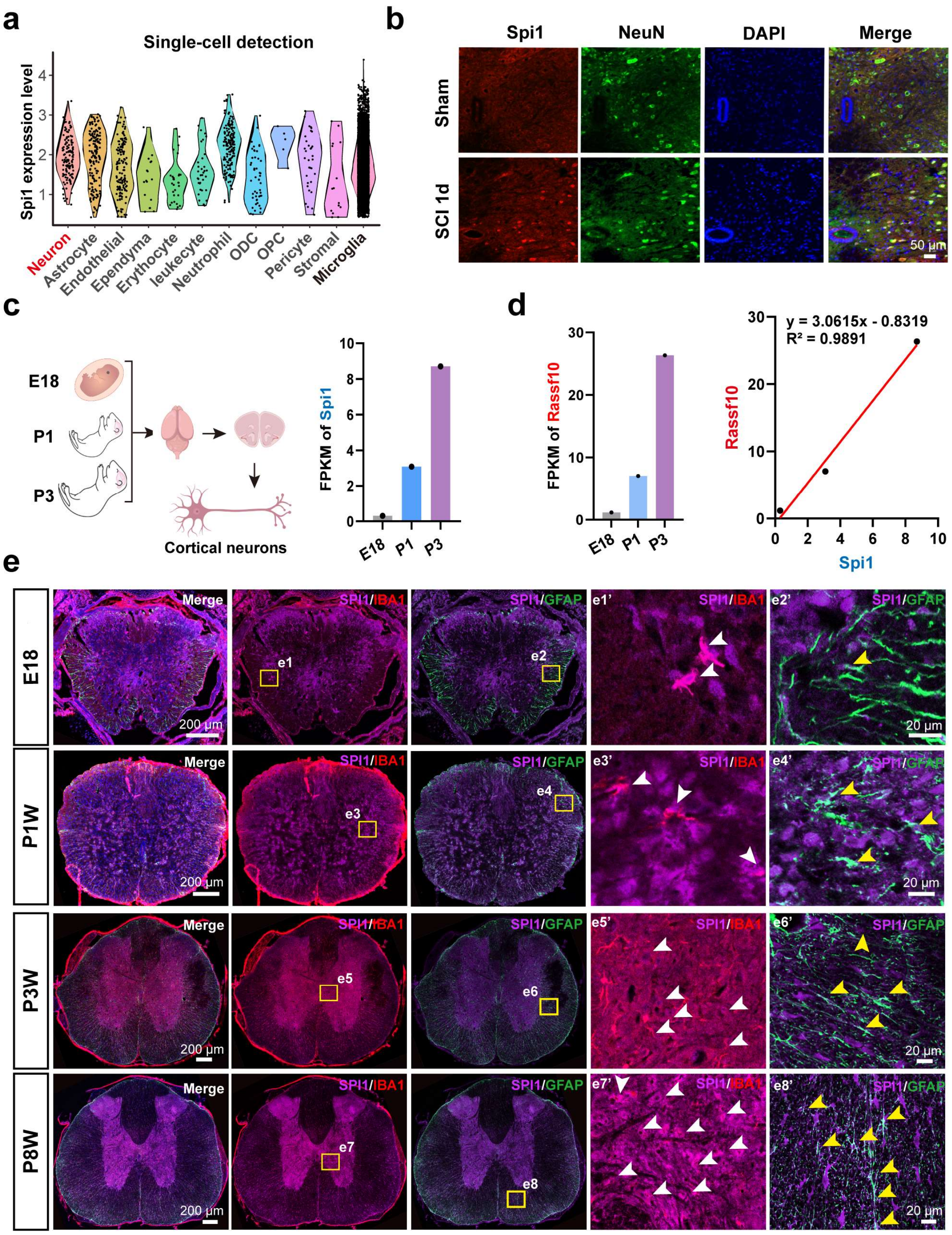

Supplementary Figure S1. Expression patterns of SPI1 and Rassf10 during the E18-P1-P3 developmental process

(a) Single-cell analysis of mouse spinal cord tissue showing the expression of SPI1 in various cell types, each dot denotes an individual SPI1-expressing cell.

(b) Spinal cord injury induced elevated SPI1 expression and enhanced its nuclear translocation in neurons.

(c) Left panel: schematic illustration of the experimental workflow: cortical neurons were isolated and cultured from the cerebral cortex of E18 embryos, P1 and P3 postnatal mice. Right panel: bar

graph showing the Spi1 mRNA expression level (represented by FPKM) in cortical tissues at E18, P1, and P3 developmental stages.

(d) Left panel: bar graph showing the Rasf10 mRNA expression level (represented by FPKM) in cortical tissues at E18, P1, and P3 developmental stages. Right panel: scatter plot with linear regression analysis demonstrating a strong positive correlation between Spi1 and Rasf10 expression levels (regression equation: $y=3.0615x-0.8319$, R2 =0.9891).

(e) Immunofluorescence staining of spinal cord cross-sections from E18, P1W, P3W, and P8W rats co-labeled with Spi1 (purple) and the microglial marker IBA1 (red) or astrocytic marker GFAP (green). Merged images (leftmost panels) show overall tissue architecture. Magnified views (e1'-e8') of the boxed regions (e1–e8) highlight Spi1 co-localization with IBA1 (white arrowheads) and GFAP (yellow arrowheads) at different developmental stages. Scale bars = 200 μm (low-magnification views), 20 μm (high-magnification views).

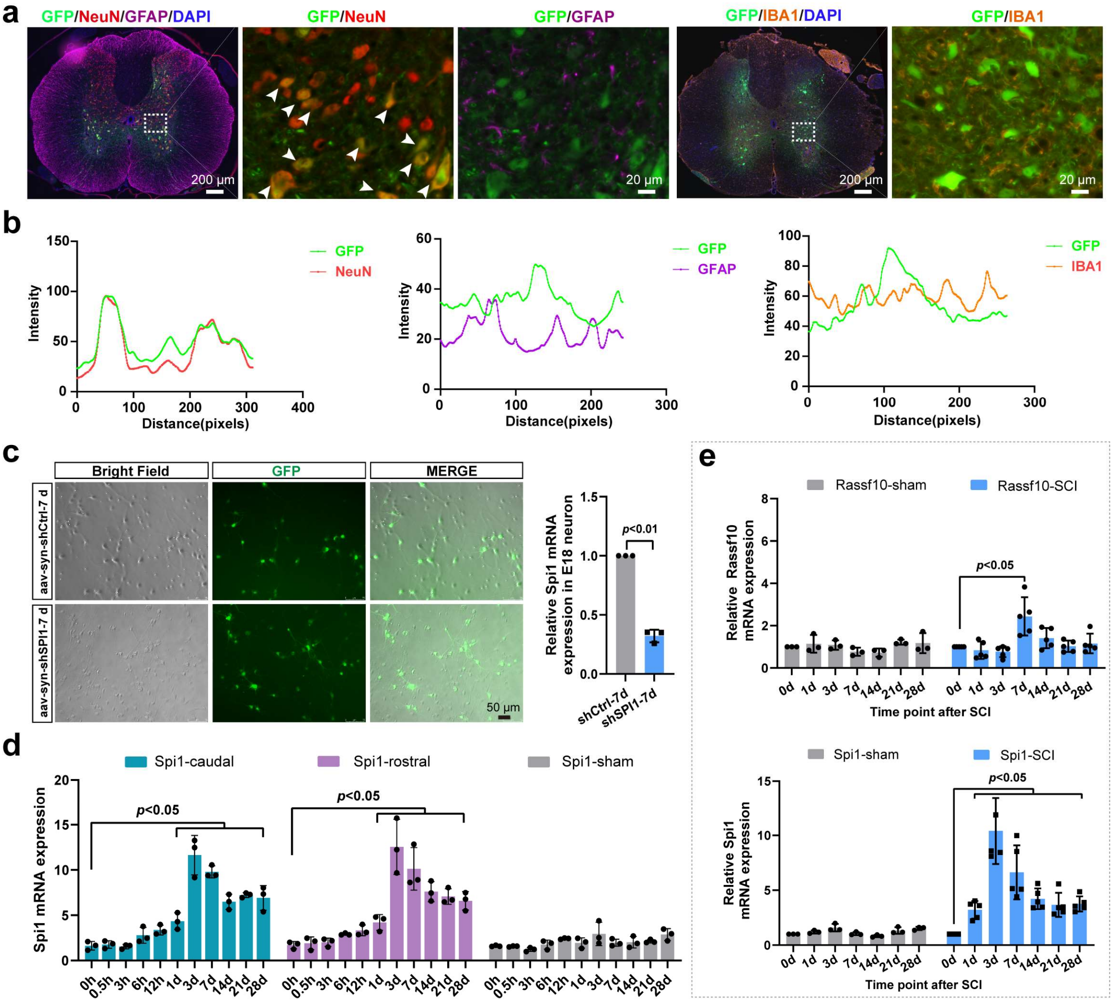

Supplementary Figure S2. Knockdown efficiency of SPI1 virus and SPI1 expression changes at various time points after spinal cord injury

(a) Representative immunofluorescence images of spinal cord sections showing GFP expression driven by the synapsin promoter. Co-staining with NeuN (neurons), GFAP (astrocytes), and IBA1 (microglia) confirms the cell-type specificity of AAV-syn-GFP transduction. Dashed boxes indicate regions magnified in the right panels. Scale bars = 200 μm (low magnification), 20 μm (high magnification).

(b) Fluorescence intensity line profiles of GFP with NeuN, GFAP and IBA1.

(c) Left panel: representative bright field, GFP fluorescence, and merged images of E14 spinal neurons 7 days after transduction with AAV-syn-shCtrl (control) or AAV-syn-shSPI1 (Spi1 knockdown) vectors. Scale bar = 50 μm. Right panel: quantitative analysis of relative Spi1 mRNA expression in E14 neurons 7 days after transduction with shCtrl or shSPI1 vectors ($p < 0.01$, Student's t-test).

(d) Sequencing data show that dynamic changes in Spi1 mRNA expression at different time points (0 h, 0.5 h, 3 h, 6 h, 12 h, 1 d, 3 d, 7 d, 14 d, 21 d, 28 d) after spinal cord injury. Statistical significance was determined by one-way ANOVA ($p < 0.05$).

(e) qPCR validation of dynamic changes in Rassf10 (a) and Spi1 (b) mRNA expression at different time points (0 d, 1 d, 3 d, 7 d, 14 d, 21 d, 28 d) after spinal cord injury. Statistical significance was determined by one-way ANOVA ($p < 0.05$).

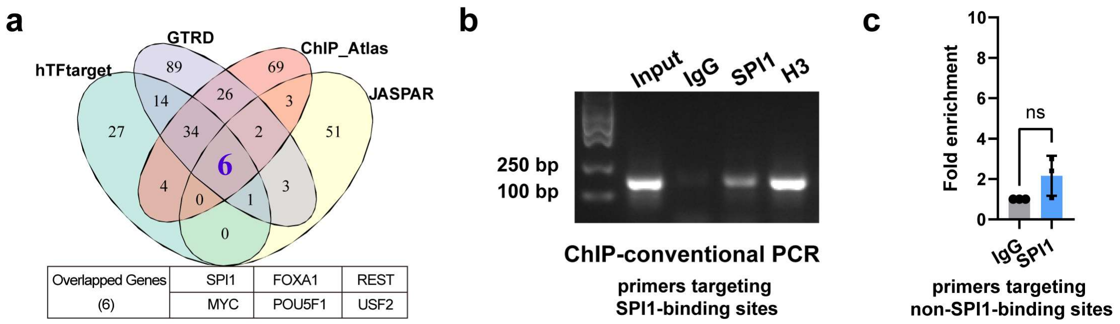

Supplementary Figure S3. Prediction of transcription factors binding to the Rassf10 promoter and ChIP assay results

(a) Venn diagram showing the overlap of potential transcription factors (TFs) predicted to bind the RASSF10 promoter region, derived from four databases (hTFtarget, GTRD, ChIP_Atlas, JASPAR). The intersection of all datasets identified 6 core candidate TFs, including SPI1, MYC, FOXA1, POU5F1, REST, and USF2.

(b) Conventional ChIP-PCR validation of SPI1 binding to its predicted site in the RASSF10 promoter. Input served as the positive control for template DNA, IgG as the negative control for non-specific binding, H3 as the positive control for chromatin immunoprecipitation efficiency, and SPI1 as the experimental group.

(c) Quantitative ChIP-qPCR analysis showing the fold enrichment of non-SPI1-binding sites in the RASSF10 promoter (ns, not significant; Student's t-test).

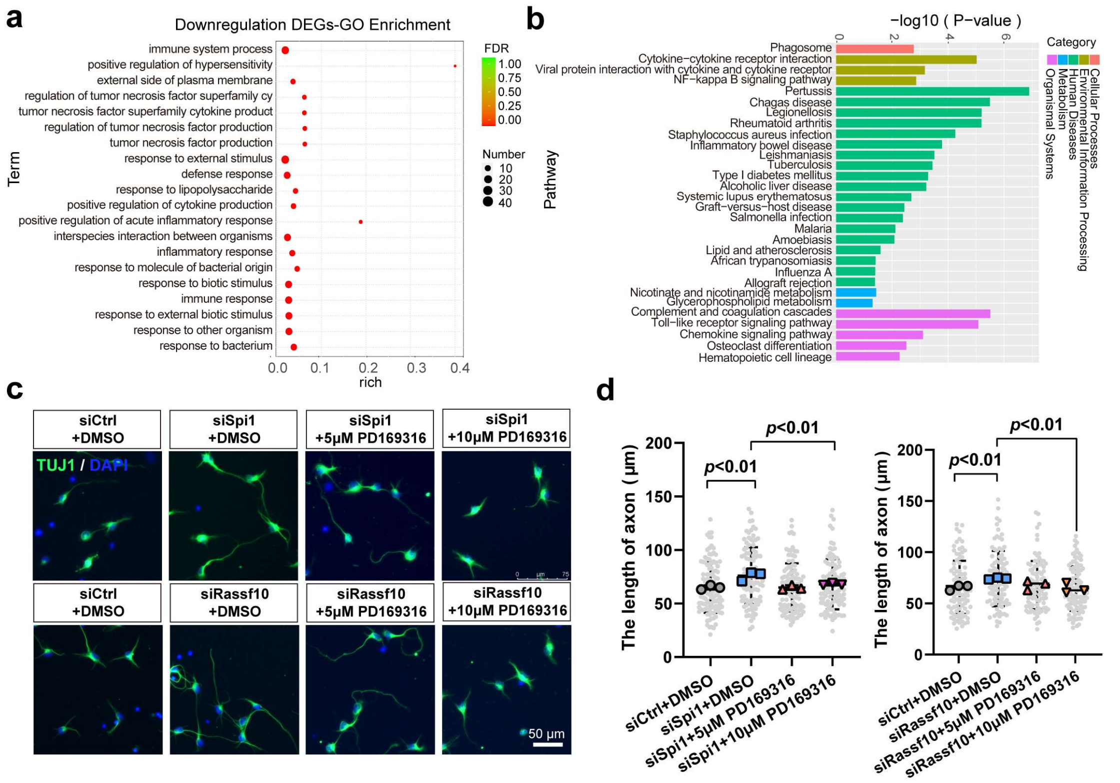

Supplementary Figure S4. GO and KEGG enrichment analysis of DEGs following SPI1 knockdown and impact of PD169316 (p-P38 MAPK specific inhibitor) on axonal growth after Spi1/Rassf10 siRNA treatment.

(a) GO enrichment analysis of downregulated DEGs. Immune and inflammatory-related biological processes, including "immune system process," "inflammatory response," and "positive regulation of hypersensitivity," were prominently enriched.

(b) KEGG pathway enrichment analysis of downregulated DEGs. Significantly enriched pathways included "Phagosome," "Cytokine–cytokine receptor interaction," and "NF-kappa B signaling pathway," indicating that SPI1 knockdown affects immune and inflammatory responses.

(c) The representative IF images of Tuj1 treated with PD169316 (5 μM, 10 μM) for 48 h in neurons with a scale bar of 50 μm.

(d) Statistical analysis. Data are presented as mean ± SD, n = 3 biological replicates, the grey dots represent 90 neurons. Unpaired Student's t-test was used to compare two groups (vs. siCtrl+DMSO). The *p*-values are shown on the panel.
